# pH/GSH dual-responsive nanoparticle for auto-amplified tumor therapy of breast cancer

**DOI:** 10.1186/s12951-024-02588-0

**Published:** 2024-06-10

**Authors:** Shengnan Huang, Zhiling Xu, Weiwei Zhi, Yijing Li, Yurong Hu, Fengqin Zhao, Xiali Zhu, Mingsan Miao, Yongyan Jia

**Affiliations:** 1 Academy of Chinese Medical Sciences, Henan University of Chinese Medicine, Zhengzhou, Henan Province 450046 P. R. China; 2 School of Pharmaceutical Sciences, Key Laboratory of Targeting Therapy and Diagnosis for Critical Diseases, Zhengzhou University, Zhengzhou, Henan Province 450001 P.R. China; 3 School of Pharmacy, Henan University of Chinese Medicine, Zhengzhou, Henan Province 450046 P. R. China

**Keywords:** Breast cancer, Auto-amplified tumor therapy, pH/GSH dual-responsive, GSH depletion, Reactive oxygen species

## Abstract

Breast cancer remains a malignancy that poses a serious threat to human health worldwide. Chemotherapy is one of the most widely effective cancer treatments in clinical practice, but it has some drawbacks such as poor targeting, high toxicity, numerous side effects, and susceptibility to drug resistance. For auto-amplified tumor therapy, a nanoparticle designated GDTF is prepared by wrapping gambogic acid (GA)-loaded dendritic porous silica nanoparticles (DPSNs) with a tannic acid (TA)-Fe(III) coating layer. GDTF possesses the properties of near-infrared (NIR)-enhanced and pH/glutathione (GSH) dual-responsive drug release, photothermal conversion, GSH depletion and hydroxyl radical (·OH) production. When GDTF is exposed to NIR laser irradiation, it can effectively inhibit cell proliferation and tumor growth both in vitro and in vivo with limited toxicity. This may be due to the synergistic effect of enhanced tumor accumulation, and elevated reactive oxygen species (ROS) production, GSH depletion, and TrxR activity reduction. This study highlights the enormous potential of auto-amplified tumor therapy.

**Supplementary Information:**

The online version contains supplementary material available at 10.1186/s12951-024-02588-0.

## Introduction

Breast cancer (BC) is one of the malignancies with the highest morbidity and mortality rates, seriously affecting human health and even endangering lives [1]. Although chemotherapy has been an effective cancer treatment in clinical practice, its efficacy is still restricted due to some drawbacks, such as low accumulation/retention of drugs, poor tumor targeting, adverse reactions, and multidrug resistance (MDR) [2–4]. Therefore, it is urgent to develop more effective cancer therapeutic strategies. Combination therapy has been investigated as an effective strategy to improve cancer chemotherapy because it can target various therapeutic pathways in cancer cells while using lower medication concentrations compared to mono-chemotherapy [5]. For instance, the efficacy of reactive oxygen species (ROS)-based treatments, such as photodynamic therapy (PDT), chemodynamic therapy (CDT), sonodynamic therapy (SDT), and radiotherapy (RT), are significantly improved when combined with other therapeutic modalities or strategies, such as immunotherapy, photothermal therapy (PTT), chemotherapy, glutathione (GSH) depletion and remodeling the tumor microenvironment (TME) [6–13].

Gambogic acid (GA), discovered from the traditional Chinese medication Garcinia cambogia, is an active monomer with a distinctive bridging xanthone structure [14]. Modern pharmacological research has recently shown that GA actively contributes to the development of multiple malignancies at different stages via various mechanisms, including the induction of autophagy, cell cycle arrest, tumor cell apoptosis, inhibition of tumor metastasis, and anti-angiogenesis [15, 16]. For instance, Li et al. demonstrated the cytotoxic and antimigratory effects of GA on MDA-MB-231 cells [17]. More intriguingly, GA promoted SMMC-7721 apoptosis by increasing ROS levels and GSH depletion and GA inhibited thioredoxin activity and induced ROS-mediated cell death in desmoplasia-resistant prostate cancer [18, 19]. Preliminary findings from a phase IIa exploratory study demonstrated that GA still had a positive safety profile even when given intravenously at 45 mg/m^2^ (2 weeks as a treatment course, 1–5 days of dosing, every other day for a total of 5 doses) [20]. In summary, GA is one of the most promising candidates for breast cancer treatment with high efficiency and low toxicity.

Despite its high anticancer activity, GA is a fat-soluble molecule with poor stability, rapid plasma clearance, and widespread dispersion in the body, significantly limiting its clinical application. Given the rapid development of nanotechnology and its wide application in the biomedical field, there is great potential to improve effective GA delivery through nanotechnology [4, 21]. Dendrimer-like porous silica particles (DPSNs) have garnered significant attention owing to their distinctive physicochemical characteristics. DPSNs represent a specialized category of three-dimensional (3D) nanostructures characterized by enhanced pore permeability, increased pore volume, and improved access to the inner surface of the particle [22]. The exceptional properties of 3D nanostructures hold considerable potential for biomedical applications, as they can be employed for the direct encapsulation or binding of therapeutic drugs, thereby enhancing their stability, and targeting through surface functionalization. More interestingly, Huang and Zhang et al. reported that DPSNs could enhance the efficacy of PDT and CDT by depleting GSH to elevate ROS levels [23, 24]. Furthermore, it was demonstrated that DPSNs remained biocompatible in mice even when administered doses as high as 30 mg/kg for up to 90 days [25].

The ability of antitumor drugs loaded into nanoparticles to be rapidly released at the tumor site and reach therapeutic concentration is the key to determine their antitumor efficacy. Intelligent drug delivery systems, that utlize the differences between the TME and normal physiological conditions are becoming a research focus [26–29]. Compared to traditional silicon oxide nanoparticles, DPSNs exhibit redox-triggered degradation, which is mainly attributed to the presence of tetrasulfide (-S-S-S-S-) bonds within their framework. This enabled the liberation of drugs contained in their cavities, thus conferring GSH-responsive characteristics to the DPSNs-based nano drug delivery system (NDDS). Nanoparticles that respond to dual or multiple stimuli provide increased sensitivity to external triggers and more precise control of drug delivery and release, surpassing the capabilities of NDDS that respond to a single stimulus [30]. Tannic acid (TA) has been reported to possess the capability to interact with 18 metal ions of varying valence states, resulting in the formation of a metal polyphenol network (MPN) that demonstrates pH-dependent disintegration characteristics [31, 32]. Among them, the TA-Fe(III) system has been most widely studied. TA-Fe(III) has the capability to effectively convert light energy into thermal energy upon laser exposure, enabling its application in PTT and photoacoustic imaging (PAI) [33]. Moreover, TA-Fe(III) acts as a CDT agent that catalyzes a Fenton-like reaction that generates hydroxyl radicals (•OH) to damage cancer cells [34].

**Scheme 1:**
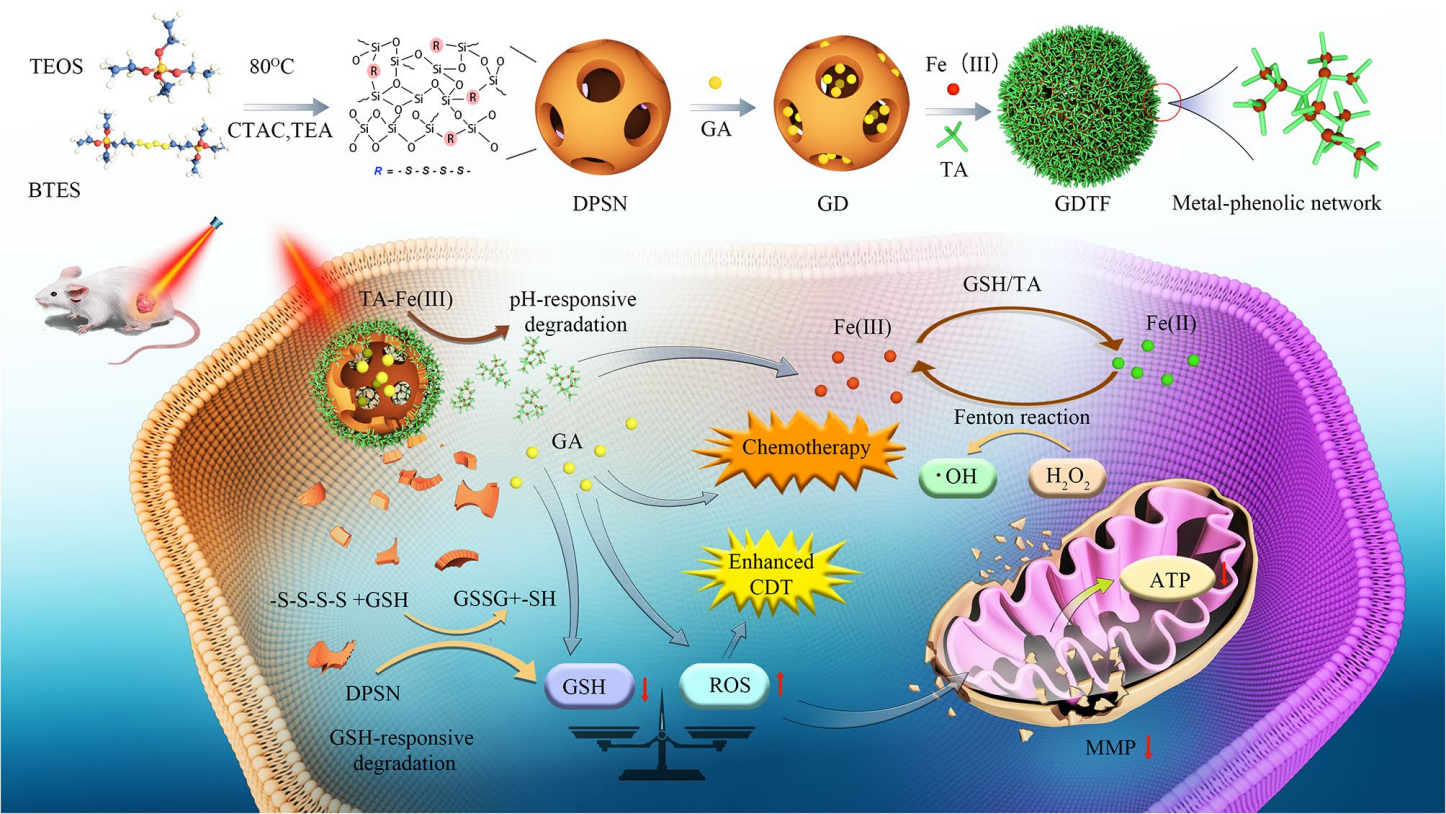
Preparation and mechanism of GDTF for auto-amplified tumor therapy. GDTF was prepared by wrapping GA-loaded DPSN with a TA-Fe(III) coating layer. GDTF had the property of pH/GSH dual-responsive degradation and drug release, and synergistically induced GSH depletion and ROS production for auto-amplified tumor therapy

Herein, GDTF was constructed by wrapping a GA-loaded DPSN in a TA-Fe(III) coating layer for auto-amplified breast cancer therapy (Scheme 1). The insolubility, stability, and drug loading capacity of GA could be significantly improved by GDTF nanoparticles. Through enhanced permeability and retention (EPR) effect, hydrophilicity and cell adhesion of TA, GDTF effectively accumulated and penetrated in the tumor site after injection. After GDTF was endocytosed into tumor cells, its TA-Fe(III) layer gradually disintegrated in the weakly acidic TME and released TA, which acted as an acid-activated reductant to reduce Fe^3+^ to Fe^2+^. Subsequently, the generated Fe^2+^ reacted with H_2_O_2_ via the Fenton reaction to generate toxic •OH, inducing cells apoptosis to achieve CDT. In addition, high intracellular GSH triggered DPSN degradation and GA release, resulting in GSH depletion and TrxR activity reduction. The heat generated by the GDTF when exposed to near infrared (NIR) laser irradiation accelerated drug release and •OH generation, achieving the chemotherapy/CDT/PTT synergistic effect. This research provided a new approach for auto-amplified cancer therapy.

## Results and discussion

**Fig. 1:**
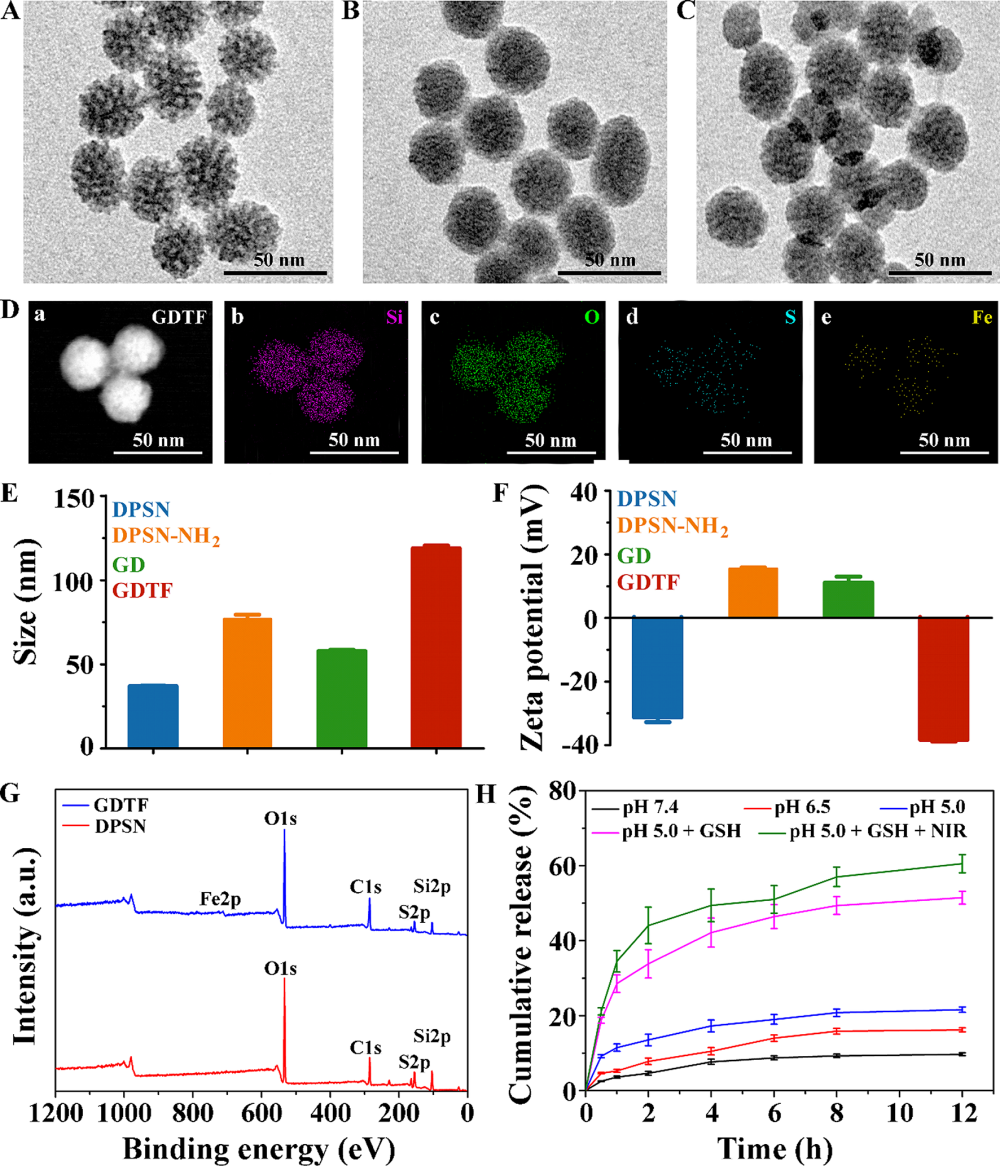
Characterization of GDTF. TEM images of (**A**) DPSN, (**B**) GD and (**C**) GDTF. The scale bar is 50 nm. (**D**) (**a**) STEM image of GDTF and (**b**–**e**) the corresponding elemental mapping images of Si, O, S and Fe. The scale bar is 50 nm. (**E**) Hydrodynamic sizes of the DPSN, DPSN-NH_2_, GD and GDTF (*n* = 3). (**F**) Zeta potential of the DPSN, DPSN-NH_2_, GD and GDTF (*n* = 3). (**G**) XPS spectra of DPSN and GDTF. (**H**) Cumulative release curves of GA from GDTF under various conditions (*n* = 3)

DPSN with dendritic 3D nanostructures was successfully synthesized as previously reported [25, 35]. To optimize the synthesis of DPSN, we examined the volume of TEOS, template remover and stirring times for template removal. The volume of TEOS displayed a negligible effect on the hydrodynamic size, polydispersity index (PDI) and zeta potential of the DPSN (Table S1). When the TEOS volume was 1.0 mL, DPSN had the smallest hydrodynamic size and PDI, as well as the highest absolute value of the zeta potential. After removal of the template by NaCl-methanol (8 mg/mL), DPSN had a smaller hydrodynamic size, smaller PDI, and higher absolute zeta potential (Table S2). The hydrodynamic size of the DPSN continued to decrease as the stirring periods for template removal lengthened, while the absolute value of the zeta potential increased (Table S3). Thus, 1.0 mL TEOS, NaCl-methanol (8 mg/mL), and five repetitions were applied in the subsequent experiments. Transmission electron microscopy (TEM) demonstrated that monodispersed spherical-like DPSN had a regular dendritic morphology, uniform size distribution, no aggregation between nanoparticles, and central radial pores (Fig. 1A). The hydrodynamic size of the DPSN tested by dynamic light scattering (DLS) was 37.01 ± 0.37 nm, which was like that observed by TEM (Fig. 1E**)**. The zeta potential of the DPSN was − 31.20 ± 1.57 mV (Fig. 1F). The nitrogen adsorption-desorption isotherm with type IV isotherm characteristics had a small hysteresis loop, demonstrating the mesoporous structure of the DPSN (Figure S1). The specific surface area, pore volume, and average pore size of the DPSN were calculated to be 522.53 ± 6.33 m²/g, 0.85 cm^3^/g and 6.47 nm respectively, which were favorable for subsequent drug loading.

Subsequently, amino-modified DPSN (DPSN-NH_2_) was prepared, and the amino content was calculated to be 0.81 mmol/g from the standard curve (Figure S2). The hydrodynamic size and zeta potential of DPSN-NH_2_ were 77.00 ± 2.56 nm and 15.50 ± 0.37 mV, respectively, demonstrating successful amino functionalization on the surface of the DPSN. Thereafter, GA-loaded DPSN (GD) was synthesized by the electrostatic interaction of DPSN-NH_2_ and GA. The encapsulation efficiency (EE, %) and drug loading (LD, %) calculated from the standard curves increased with decreasing drug amounts as the mass ratio of drug to carrier decreased from 2:1 to 1:10 (Figure S3, Table S3 and S4). EE and DL peaked at the 1:10 mass ratio of drug to carrier, where they were 97.37 ± 0.38% and 9.16 ± 0.07%, respectively. Therefore, a mass ratio of drug to carrier of 1:10 was chosen for the following experiments. The dendritic branches and central radial orifices were almost invisible in GD, which may be due to the large amount of GA loaded in the GD (Fig. 1B). The hydrodynamic size of GD was 57.87 ± 0.71 nm, which was smaller than DPSN-NH_2_ but larger than DPSN. This might be because the GD structure became compact after the electrostatic interaction of DPSN-NH_2_ and GA. The zeta potential of GD was 11.80 ± 1.88 mV and was slightly lower than that of DPSN-NH_2_, indicating that negatively charged GA was successfully loaded in GD.

To prevent drug leakage, an MPN layer consisting of TA and Fe(III) was modified on the surface of the GD nanoparticles to obtain TA-Fe(III) layer-wrapped GD (GDTF). GDTF solution was brownish-black with no obvious precipitation, delamination, flocculation, emulsification and cracking or other unstable phenomena, while GD solution was milky-yellow and DPSN solution was colorless with blue opalescence (Figure S4). TEM images revealed a similar structure of GDTF with GD with no obvious dendritic branches or central radial orifices (Fig. 1C). Compared to GD, the hydrodynamic size of GDTF was larger (118.97 ± 1.50 nm) due to the presence of TA-Fe(III) layer. The potential of GDTF nanoparticles was − 38.30 ± 0.59 mV, confirming the successful wrapping of the TA-Fe(III) layer. The results of scanning transmission electron microscopy and energy dispersive X-ray spectroscopy (STEM-EDX) mapping displayed uniform compositional distributions of the four elements (Si, O, S and Fe) in GDTF, suggesting a sphere-like structure and successful coating of TA-Fe(III) layer (Fig. 1D). The results of X-ray photoelectron spectroscopy (XPS) revealed that both DPSN and GDTF had four elements (O, C, S and Si), but GDTF also contained Fe, which was consistent with the results of STEM-EDX mapping (Fig. 1E). According to the standard curve of the inductively coupled plasma-optical emission spectrometer (ICP-OES), it was determined that GDTF contained 149.68 mg/L of Fe (Figure S5). The successful synthesis of GDTF was also confirmed by FT-IR experiment (Figure S6). Significantly, FT-IR spectra of GDTF contained similar peaks with DPSN, such as Si-O-Si symmetric stretching vibration absorption peak at 795 cm^− 1^, and Si-O-Si anti-symmetric stretching vibration absorption peak at 1046 cm^− 1^. FT-IR spectra of GDTF contained the same C = O vibration absorption peak at 1690 cm^− 1^ with GA, while had a slight blueshift of O-H bending vibration absorption peak at 1344 cm^− 1^ from 1331 cm^− 1^ in GA. This may ascribe to the -COOH bond of GA was conjugated on the -NH_2_ bond on the surface of DPSN-NH_2_. FT-IR spectra of GDTF contained the same C = O and C = C tensile vibration peaks at 1690 cm^− 1^ and 1605 cm^− 1^, respectively. Compared to the wide O-H tensile vibration peaks at 3264 cm^− 1^ of TA, GDTF had weak peak, attributing to the coordination of multiple phenolic hydroxyl groups in TA with metallic iron.

**Fig. 2:**
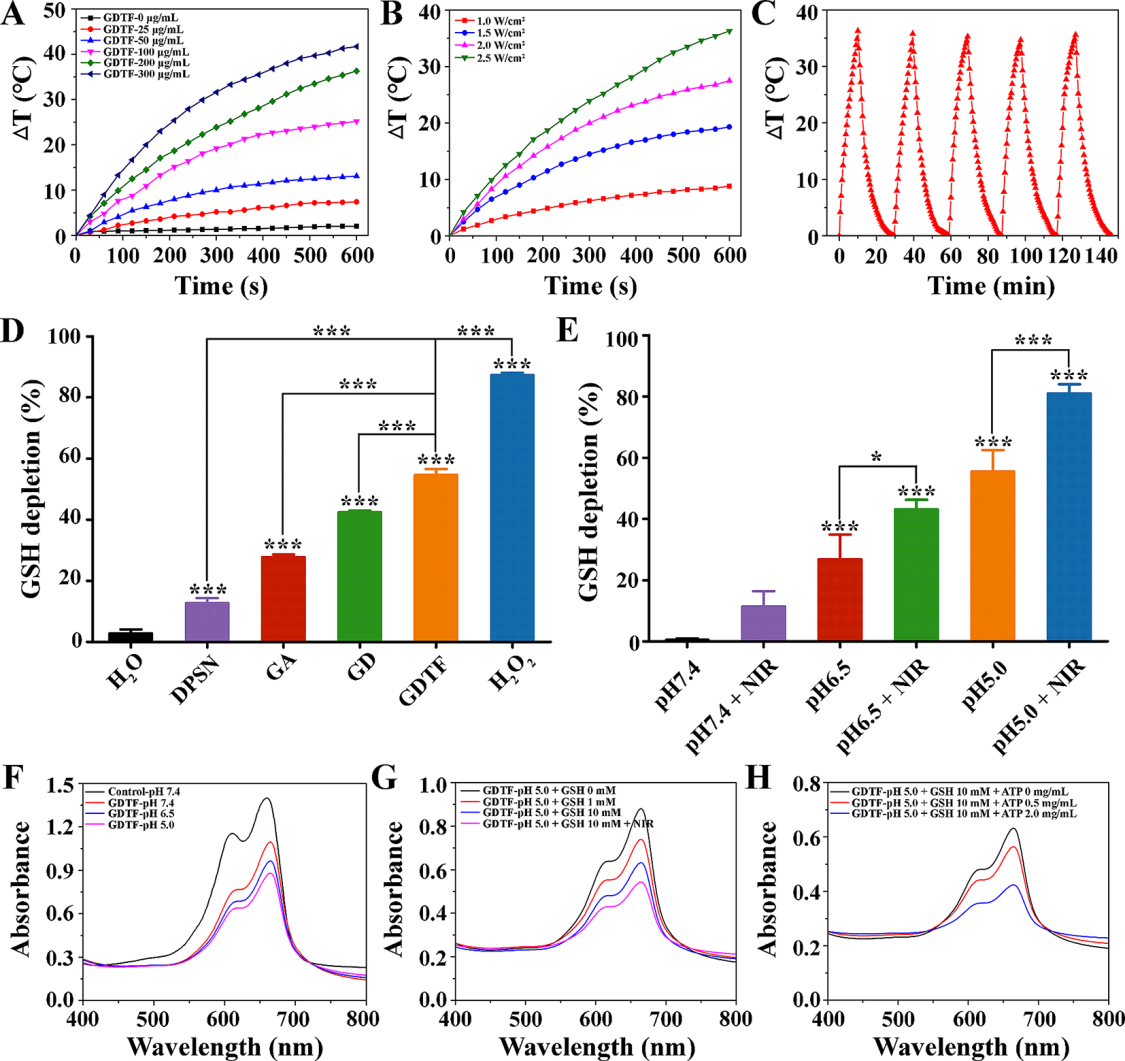
Photothermal conversion capacity, the ability of GSH depletion and •OH generation of GDTF. (**A**) Temperature changes of GDTF at various concentrations (0, 25, 50, 100, 200 and 300 µg/mL) when exposed to 808 nm laser irradiation at a power density of 2.5 W/cm^2^ for 600 s. (**B**) Temperature changes of GDTF at 400 µg/mL when exposed to 808 nm laser irradiation at various power densities (1.0, 1.5, 2.0 and 2.5 W/cm^2^) for 600 s. (**C**) Temperature changes of GDTF at 400 µg/mL when exposed to 808 nm laser irradiation at a power density of 2.5 W/cm^2^ during 5 ON/OFF cycles. (**D**) GSH depletion of H_2_O (negative control), DPSN, GA, GD, GDTF and H_2_O_2_ (positive control) (*n* = 3). ^***^P < 0.001, significantly different from H_2_O and GDTF. (**E**) GSH depletion of GDTF at pH 7.4, 6.5 and 5.0 with and without NIR irradiation (*n* = 3). ^*^P < 0.05 and ^***^P < 0.001, significantly different from GDTF at pH 7.4 and NIR irradiation. (**F**) The change in the UV‒vis‒NIR spectra of MB over reaction time during the Fenton-like reaction triggered by GDTF at pH 7.4, 6.5 and 5.0. (**G**) The change in the UV–vis–NIR spectra of MB over reaction time during the Fenton like reaction triggered by GDTF at pH 5.0 in the presence of GSH at various concentrations (0, 1 and 10 mM) when exposed to NIR irradiation. (**H**) The change in the UV–vis–NIR spectra of MB over reaction time during the Fenton-like reaction triggered by GDTF at pH 5.0, with the presence of 10 mM GSH and ATP at various concentrations (0, 0.5 and 2.0 mg/mL)

The temperature rise of GDTF was positively correlated with its concentration, laser power density and irradiation time, indicating that GDTF had good photothermal conversion capacity (Fig. 2A and B and S7). This meant that GDTF temperature could be adjusted by changing the solution concentration or laser power density, thus facilitating the subsequent cancer treatment. The temperature change curves during 5 on/off laser irradiations indicated the stable photothermal conversion capacity of GDTF (Fig. 2C).

Since drug release behavior in TME was crucial for subsequent tumor therapy, we investigated the effects of pH, GSH, and NIR laser irradiation on GA release from GDTF. Owing to the relative stability at pH 7.4, only 9.68 ± 0.39% of GA was prematurely released from GDTF within 12 h (Fig. 1H). This indicated that GA was less likely to be prematurely released from GDTF in vivo. At pH 6.5 and pH 5.0, 16.20 ± 0.54% and 21.58 ± 0.73% GA was released from GDTF within 12 h, respectively. This was slightly higher than that at pH 7.4, which might ascribe to the pH-responsive degradation of the TA-Fe(III) layer. When 10 mM GSH was added to release medium at pH 5.0, GA release rose to 51.45 ± 1.71%, which was 2.38 times higher than that without GSH. The same trend was observed when GSH was added at pH 7.4 (2.22-fold) and pH 6.5 (2.16-fold) (Figure S8). GA release further increased to 60.54 ± 2.42% when release medium at pH 5.0 containing GSH was exposed to NIR laser irradiation, which was slightly higher than that of the group without laser irradiation. GA release was triggered by the pH-sensitive rupture of TA-Fe(III) layer and GSH-responsive degradation of DPSN, and further enhanced by NIR laser irradiation, laying a good foundation for subsequent cancer therapy.

The results of 5,5’-Dithiobis-2-nitrobenzoic acid (DTNB) assay demonstrated that both the carrier DPSNs and the drug GA had the capacity to deplete GSH (Fig. 2D). As expected, the GD-mediated GSH depletion was higher compared with DPSN and GA. Moreover, GDTF had superior GSH depletion than GD because the released less active Fe(III) ions were converted to more active Fe(II) ions by TA and GSH. Weakly acidic and NIR laser irradiation further improved GSH depletion of GDTF (Fig. 2E). It should be highlighted that GDTF-mediated GSH depletion might elevate intracellular ROS levels, eventually inducing tumor cell death.

In the presence of H_2_O_2_, once GDTF was mixed with MB, the absorbance of MB at 665 nm decreased significantly, suggesting that •OH radicals were being generated (Fig. 2F). Weak acids, GSH, and NIR irradiation greatly increased the •OH generation of GDTF as more Fe(III) ions were released from GDTF, and the released less reactive Fe(III) ions were continuously converted to more reactive Fe(II) ions (Fig. 2G). In addition, ATP enhanced the •OH generation of GDTF, which might be attributed to the strong chelation of Fe with ATP leading to the degradation of the TA-Fe(III) layer by ATP (Fig. 2H) [36]. Importantly, increased ATP expression is a frequent occurrence that only affects tumor cells [37]. Thus, the entire process of •OH generation of GDTF could be summarized as follows: (1) the release of Fe(III) ions from GDTF were stimulated by weak acid, GSH and ATP, (2) the released less active Fe(III) ions could be reduced to more active Fe(II) ions by GSH and TA, and (3) the reduced Fe(II) ions more actively catalyzed H_2_O_2_ to generate •OH radicals, and were re-oxidized to Fe(III) ions, which could be reduced again by TA and GSH for additional •OH generation [38]. In summary, the •OH generation of GDTF could be performed in the TME with weak acid and elevated GSH and ATP, which endowed it with tumor-specific and NIR-enhanced •OH generation for improved tumor therapy. There were only minor changes in the particle size and EE% even after maintaining for 30 days at room temperature, demonstrating the good stability of GDTF (Figure S9). Even at the highest concentration of 500 µg/mL, the hemolysis rate of GDTF was less than 2%, indicating the high biocompatibility of GDTF (Figure S10).

**Fig. 3:**
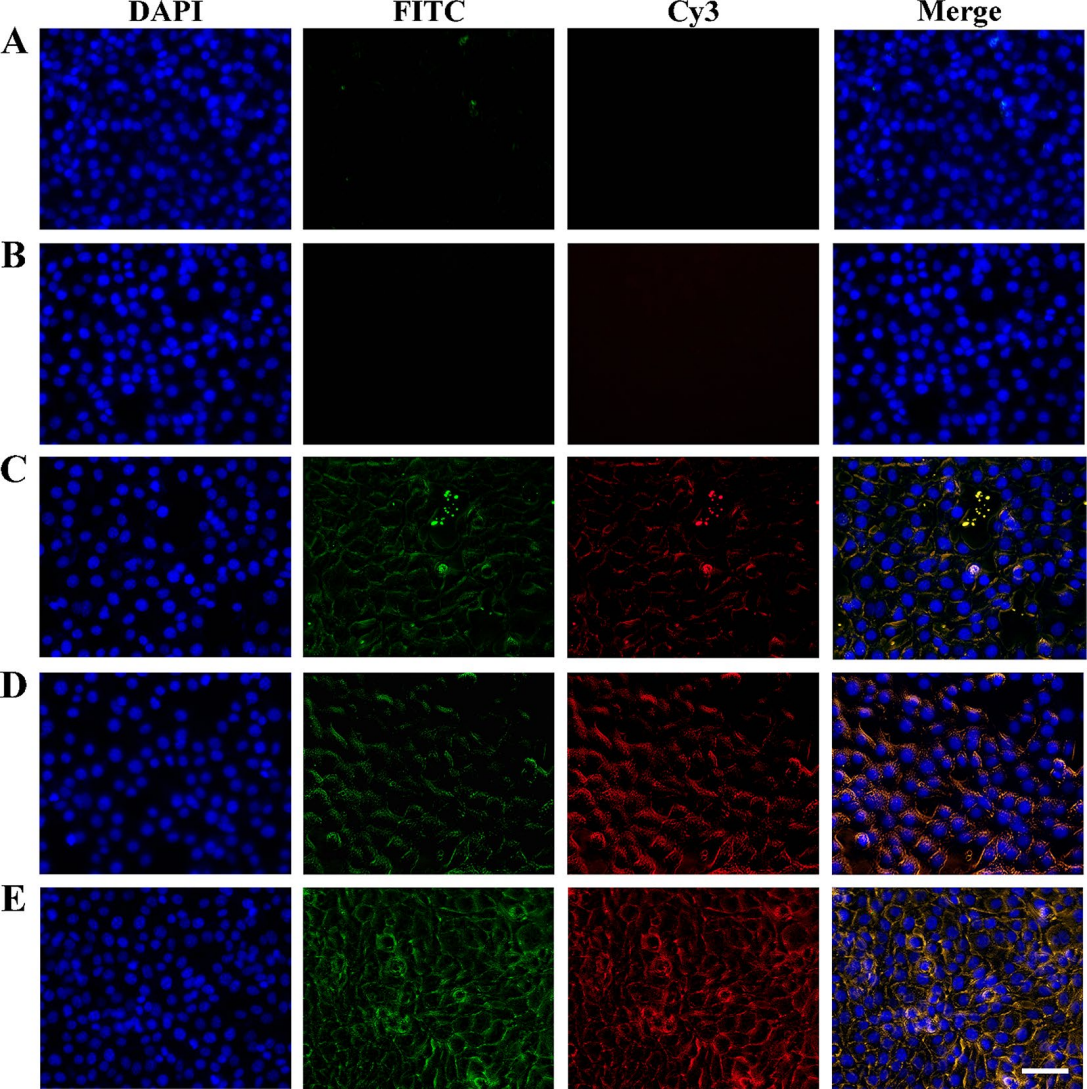
Cellular uptake of (**A**) FITC, (**B**) Cy3, (**C**) CFD, (**D**) CFDTF, and (**E**) CFDTF + NIR in 4T1 cells for 4 h. For Group E, the cells were incubated with CFDTF for 4 h and then exposed to 808 nm laser irradiation at a power density of 2.5 W/cm^2^ for 10 min. The scale bar is 50 μm

To observe the cellular uptake behavior of GDTF in 4T1 cells using fluorescence microscopy, FITC- and Cy3-loaded TA-Fe(III) layer-wrapped DPSNs (CFDTF) were prepared. The associated green and red fluorescence was difficult to detect after co-incubation of cells with uncoated FITC and Cy3, demonstrating their little cellular uptake (Fig. 3A and B). After incubation with FITC- and Cy3-loaded DPSNs (CFD), the cells displayed green and red fluorescence, demonstrating they were efficiently taken up by the cells (Fig. 3C). The cellular uptake of CFD and CFDTF groups was time-dependent, as evidenced by the brightening of fluorescence over time (Figure S11 to S13). The brighter fluorescence in the CFDTF group compared to the CFD group may be due to its strong adhesion to cells and pH-responsive degradation (Fig. 3D). The fluorescence of CFDTF + NIR group was stronger than that of the CFDTF group, which might be due to the improved permeability of the cell membrane and accelerated FITC and Cy3 release by the photothermal conversion capacity of TA-Fe(III) coating layer (Fig. 3E). The quantitative analysis results of cellular uptake in 4T1 cells measured using UV–vis–NIR spectrophotometry demonstrated the same trend as observed by fluorescence microscopy, and exhibited similar cellular uptake as MCF-7 cells (Figure S14).

**Fig. 4:**
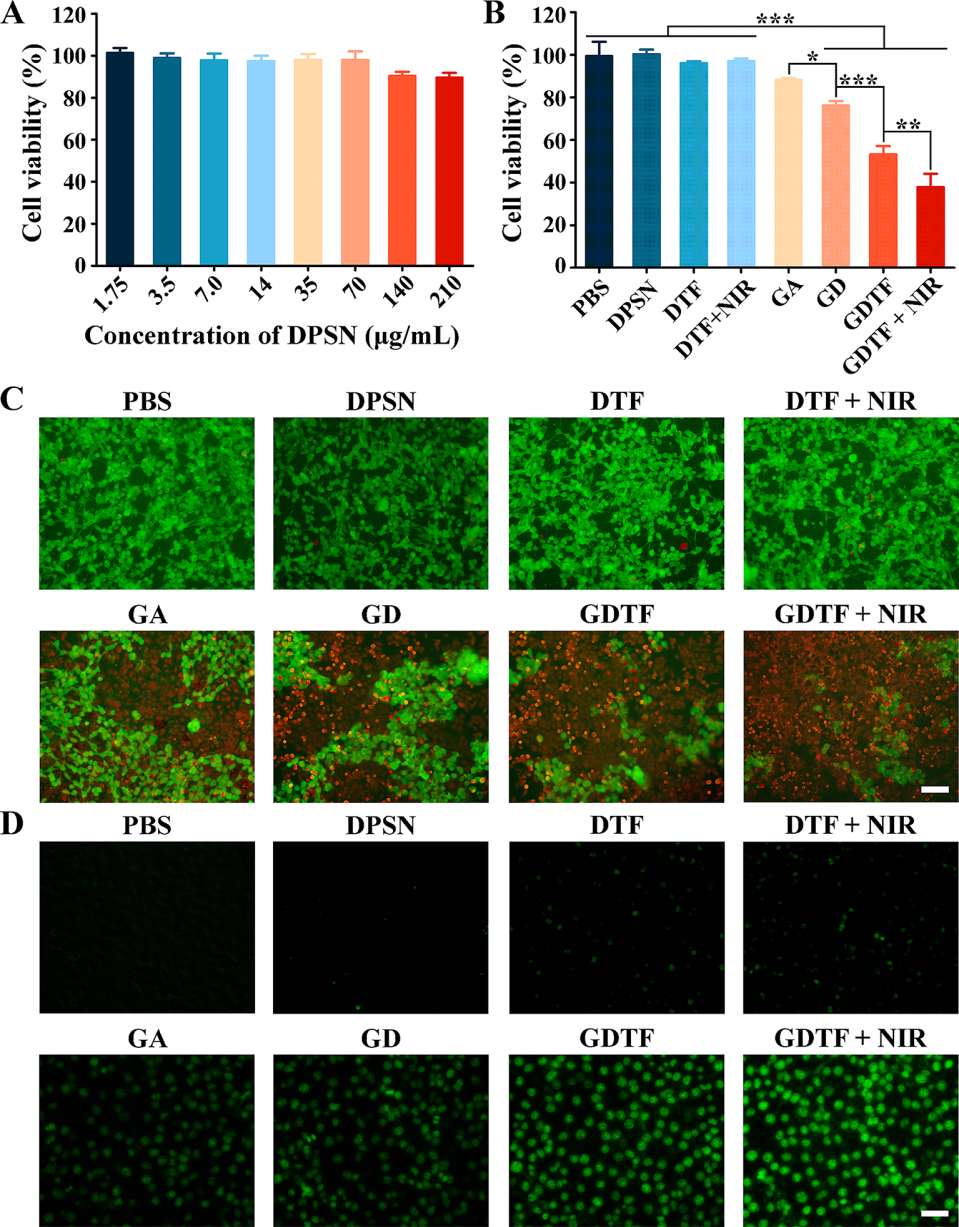
Cell viability, live/dead double staining, and intracellular ROS generation of GDTF in 4T1 cells. (**A**) Cell viability of the cells after incubation with DPSN for 48 h tested by CCK-8 assay. (**B**) Cell viability of the cells after incubation with PBS, DPSN, DTF, GA, GD and GDTF (final GA concentration was 5 µM) for 24 h. After incubation with DTF and GDTF for 4 h, the cells were exposed to 808 nm laser irradiation at a power density of 2.5 W/cm^2^ for 10 min and then incubated for 20 h. ^*^P < 0.05, ^**^P < 0.01 and ^***^P < 0.001, significantly different. (**C**) Calcein AM/PI live/dead double staining and (**D**) intracellular ROS production of the cells after incubation with PBS, DPSN, DTF, GA, GD and GDTF. The scale bar is 50 μm

The cell viability was above 90% after 48 h of incubation with DPSNs at various concentrations, demonstrating their negligible cytotoxicity (Fig. 4A). When the cells were incubated with DTF and exposed to NIR laser irradiation, their viability was slightly decreased (Fig. 4B). This might be due to the enhanced cellular uptake and Fenton-like reaction by NIR laser irradiation. GA efficiently inhibited the growth and proliferation of 4T1 cells, and this effect was significantly improved by GD, which may be benefited from the synergistic effect of enhanced cellular uptake, GSH-responsive GA release, and GSH depletion. GDTF and GDTF + NIR further enhanced the cytotoxicity through enhancing the cellular uptake, Fenton-like reaction and GSH depletion by TA-Fe(III) coating layer especially when exposed to NIR laser irradiation. Green fluorescence was more prominent in the control, DPSN, DTF, and DTF + NIR groups, while red fluorescence was barely noticeable (Fig. 4C). This indicated that there was almost no cell apoptosis after 24 h of incubation with DTF even when exposed to laser irradiation. Compared to GA, GD exhibited higher red fluorescence, indicating increased cell apoptosis. The ratio of cell apoptosis in GDTF group was higher than that in GD group, suggesting a stronger cell-killing efficacy. The highest apoptosis rate was observed in GDTF + NIR group. Moreover, the results of CCK-8 assay and live/dead staining confirmed that GDTF + NIR exhibited high anti-tumor efficacy on MCF-7 cells as well (Figure S15 to 16).

The intracellular ROS generation results determined by DCFH-DA probe displayed the same general trend as the live/dead double staining (Fig. 4D and S17). Green fluorescence was observed in DTF and DTF + NIR groups, suggesting that ROS generation may be due to Fenton-like reaction mediated by TA-Fe(III) coating. Consistent with previous findings, green fluorescence was evident in the GA group, suggesting that GA caused ROS generation [39, 40]. The higher ROS generation in GD than GA was due to a combination of increased cellular uptake and higher GSH depletion. Moreover, GDTF produced more ROS than GD, which might result from the combined effects of greater cellular uptake, higher GSH depletion, and the improved Fenton-like reaction mediated by the TA-Fe(III) layer. Improved intracellular ROS generation was exhibited when GDTF was exposed to NIR laser irradiation. These findings indicated that the trend of ROS generation was consistent with cytotoxicity results, indicating a potential direct relationship between cytotoxicity and ROS generation.

**Fig. 5:**
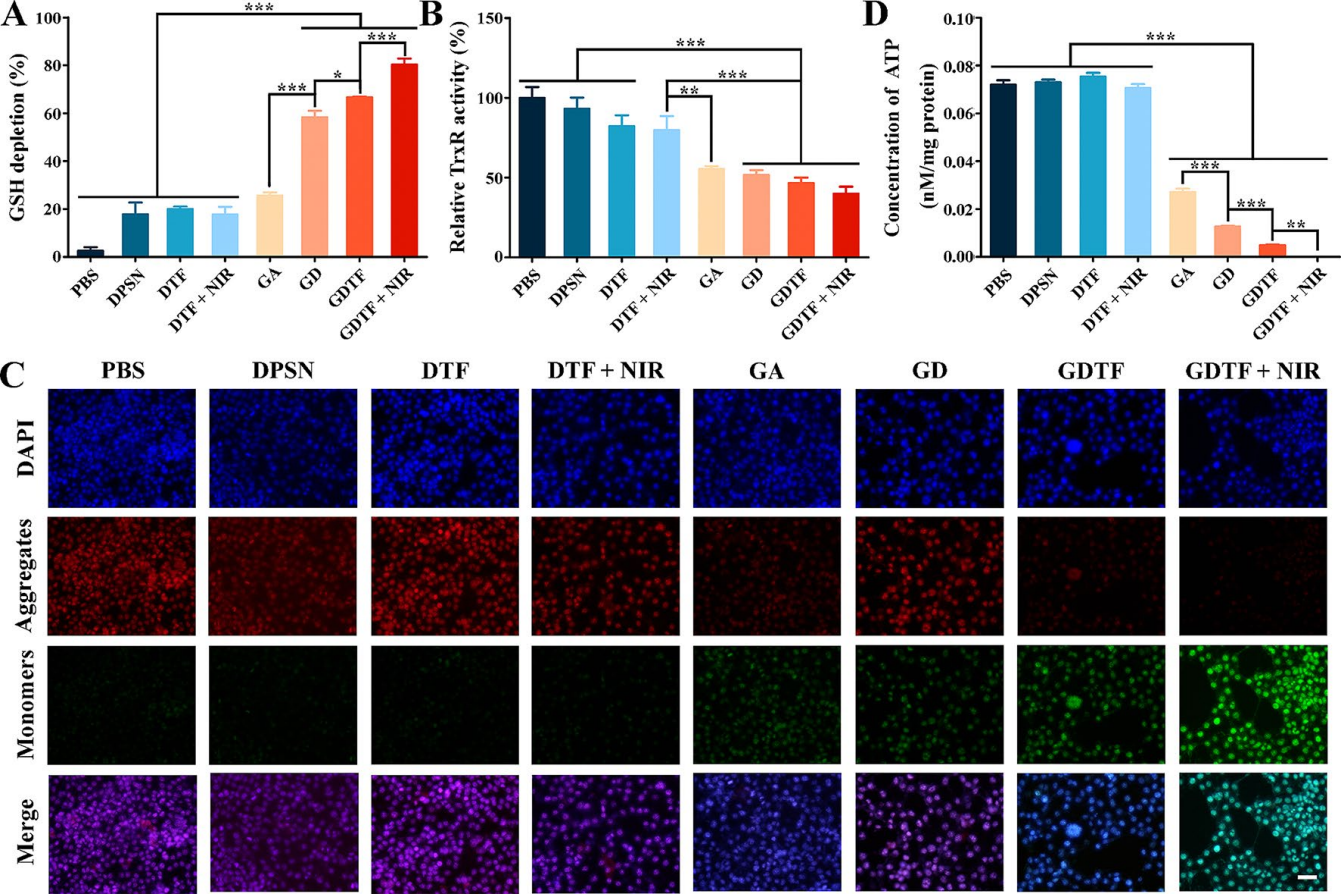
Anti-tumor mechanism of GDTF in vitro. (**A**) GSH depletion, (**B**) relative TrxR activity, and (**D**) intracellular ATP content of the cells after incubation with PBS, DPSN, DTF, GA, GD and GDTF (the final GA concentration was 5 µM) for 24 h. ^*^P < 0.05, ^**^P < 0.01 and ^***^P < 0.001, significantly different. (**C**) JC-1 assay for determining the MMP of the cells after incubation with PBS, DPSN, DTF, GA, GD and GDTF (the final GA concentration was 5 µM) for 24 h, respectively. The scale bar is 50 μm. After incubation with DTF and GDTF for 4 h, the cells were exposed to 808 nm laser irradiation at a power density of 2.5 W/cm^2^ for 10 min and then incubated for 20 h

As one of the most significant antioxidants, GSH could protect cells from the significant amount of ROS to maintain redox dynamic equilibrium. A potential method for treating cancer is to deplete GSH because this could lead to ROS accumulation that can harm or even kill cells and improve ROS-based cancer therapy [41–44]. GSH depletion was assessed to determine whether it was involved in the GDTF-induced intracellular ROS generation. The GSH depletion was in line with the intracellular ROS generation, i.e., the more GSH depletion, the higher intracellular ROS level (Fig. 5A). The superior GSH depletion capacity of GDTF was derived from the combinational effect of tetrasulfide bond in the DPSN structure and Fenton-like reaction mediated by TA-Fe(III) coating layer. These findings indicated that the GDTF-induced intracellular ROS elevation might be due to its superior GSH depletion ability, which led to the amplified oxidative stress and eventually induced apoptosis and cell death. GA has been reported to primarily target the selenocysteine (Sec) of thioredoxin reductase (TrxR) and inhibit its activity, causing an accumulation of intracellular ROS, disturbing redox balance, and ultimately resulting in cell apoptosis [45]. We further determined the relative TrxR activity to investigate whether GDTF-induced ROS generation and GSH depletion correlate with the relative TrxR activity. The intracellular relative TrxR activity was effectively reduced by GA and this effect was further improved by GD, GDTF and GDTF + NIR (Fig. 5B). This was accomplished by the enhanced cellular uptake, pH/GSH dual-responsive drug release, the mutual amplification of the targeting TrxR of GA, the photothermal conversion ability and the Fenton-like reaction of the TA-Fe(III) coating layer. This in turn amplified the intracellular oxidative stress, disrupted redox balance, and eventually induced cell apoptosis or death. In addition, high anti-tumor efficacy of GDTF + NIR in MCF-7 cells might be attributed to ROS generation, GSH depletion and TrxR activity reduction (Figure S17 to S18).

An increase in ROS can damage the mitochondrial membrane, leading to the opening of the mitochondrial membrane permeability transition pore, decreasing in the mitochondrial membrane potential (MMP), releasing cytochrome C, activating of a series of caspase enzymes, and ultimately induing cell apoptosis [46, 47]. Compared with control group and the carrier groups (DPSNs, DTF, DTF + NIR), the GA, GD, and GDTF groups showed a significant decrease in red fluorescence and a significant increase in green fluorescence, indicating a more pronounced decrease in MMP (Fig. 5C). The decrease in MMP of GDTF was more pronounced when exposed to NIR laser irradiation, as confirmed by the decreased red fluorescence and the increased green fluorescence. These results suggest that GDTF-mediated auto-amplified therapy successfully decreased MMP, which might influence the energy transport of tumor cells and hence inhibit their growth and proliferation.

As mitochondria served as the site for ATP synthesis and the decreased MMP might result in decreased ATP synthesis, the intracellular ATP content was further detected. Compared with the control group, the intracellular ATP content was dramatically decreased in the GA, GD, GDTF, and GDTF + NIR groups, whereas no significant difference was observed in the DPSNs, DTF, and DTF + NIR groups (Fig. 5D). These findings demonstrated that GDTF reduced ATP synthesis, decreased the energy supply to the cells, and eventually induced cell apoptosis. This was because that GDTF induced intracellular ROS generation, MMP decrease and caused mitochondrial malfunction. Notably, TA-Fe(III) coating has been reported to consume ATP by effectively binding Fe(III) to ATP through metal ion-triphosphate coordination [36, 38, 48].

**Fig. 6:**
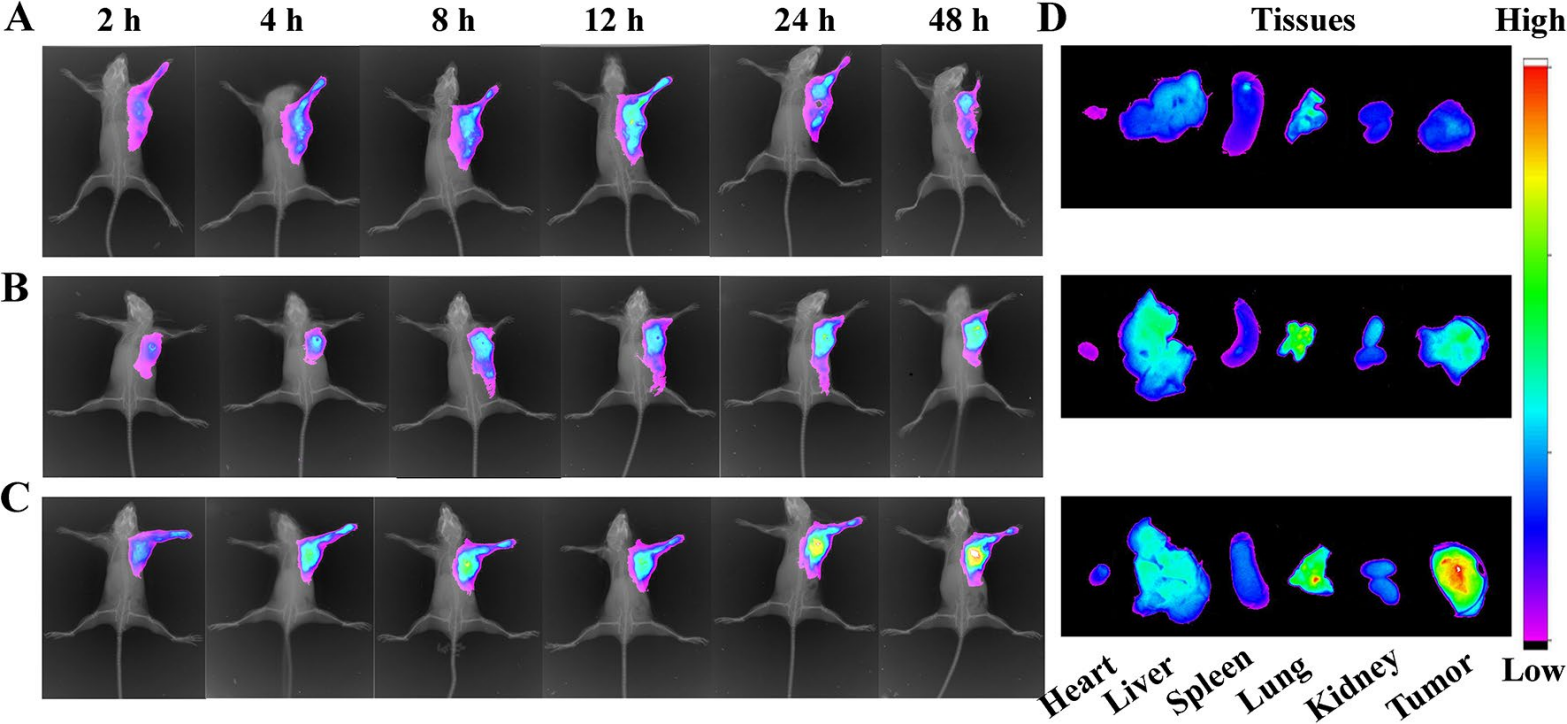
Biodistribution of GDTF in vivo. The mice were injected with (**A**) IR780, (**B**) ID, and (**C**) IDTF at an IR780 concentration of 2.0 mg/kg via the tail vein. (**D**) Biodistribution of IR780 in all groups of the main tissues, including heart, liver, spleen, lung, kidney, and tumor, at 48 h postinjection

Effective tumor accumulation was vital for subsequent tumor therapy in vivo. To determine the biodistribution of GDTF, IR780-labelled DPSN (ID) and IR780-labelled DTF (IDTF) was prepared. Tumor site accumulation of uncoated IR780 reached its peak at 12 h postinjection (Fig. 6A). Compared with IR780, ID had higher accumulation in the tumor site and reached its peak at 48 h postinjection, which might be due to the EPR effect (Fig. 6B). In addition, IDTF further improved tumor accumulation while reducing distribution in the liver, which was consistent with the research of Shim et al. [49]. This might ascribe to the reduced adsorption of serum proteins and the decreased uptake of IDTF by macrophages in the liver mediated by TA with hydrophilicity (Fig. 6C). The improved tumor accumulation of IDTF was confirmed by the biodistribution of IR780 in all groups of major tissues, including heart, liver, spleen, lung, kidney, and tumor, at 48 h postinjection (Fig. 6D). In summary, IDTF enhanced tumor accumulation through the EPR effect and the hydrophilicity of TA.

**Fig. 7:**
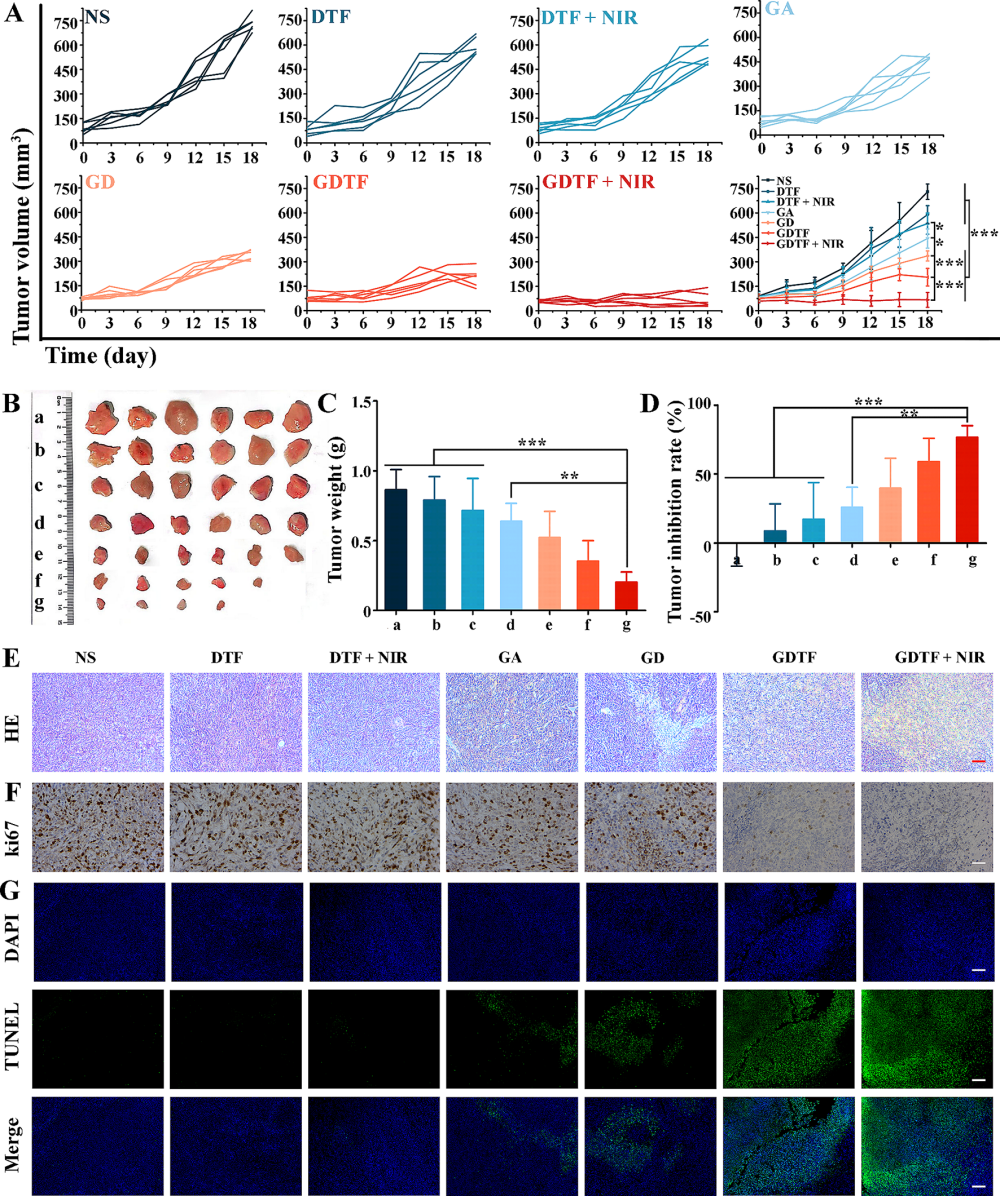
Antitumor efficacy of GDTF in vivo. (**A**) Tumor growth curves of the mice during treatments with NS, DTF, DTF + NIR, GA, GD, GDTF and GDTF + NIR at a GA concentration of 2.0 mg/kg once every three days for a total of five times (*n* = 6). The mice in the DTF + NIR and GDTF + NIR groups were exposed to an 808 nm laser at a power density of 1.0 W/cm^2^ for 5 min at 4 h postinjection. ^*^P < 0.05 and ^***^P < 0.001, significantly different. (**B**) Photographs, (**C**) tumor weight, and (**D**) tumor inhibition rate (%) of the tumors from each group. Groups a-g were NS, DTF, DTF + NIR, GA, GD, GDTF and GDTF + NIR, respectively. ^**^P < 0.01 and ^***^P < 0.001, significantly different. (**E**) HE staining, (**F**) ki67 staining and (**G**) TUNEL assay of the tumors in each group. The scale bar is 100 μm

The temperature of tumors in the NS group remained almost unchanged, while that in the GDTF group increased significantly. The temperatures of tumors in GDTF group after irradiation for 1 min and 5 min were 37.5 ℃ and 46.9 ℃, respectively, while those in NS group were 33.9 ℃ and 36.5 ℃, respectively (Figure S19). This indicated that the GDTF exhibited relatively high photothermal conversion efficacy in vivo and the great potential for PTT-based combination therapy. Inspired by the high cytotoxicity of GDTF in vitro, its antitumor effect in 4T1 tumor-bearing mice was further evaluated. Overall tumor growth in the mice was moderate for the first six days, with small differences between groups, which became more pronounced as the treatment extended **(**Fig. 7A**)**. Compared with NS, DTF and DTF + NIR slightly inhibited tumor growth due to the NIR-enhanced Fenton-like reaction mediated by the TA-Fe(III) coating layer. GA and GD moderately inhibited tumor growth, resulting from GA-mediated chemotherapy and enhanced GA delivery. In addition, GDTF inhibited tumor growth more effectively, which might be attributed to the enhanced tumor accumulation and the enhanced chemotherapy with the assistance of Fenton-like reaction mediated by the TA-Fe(III) layer. Moreover, the further improved tumor growth inhibition of GDTF when exposed to NIR laser irradiation was attributed to the enhanced tumor accumulation, drug release and Fenton-like reaction. The photographs, tumor weights and tumor inhibition rates (%) of the collected tumors of each group further confirmed the enhanced tumor growth inhibition of GDTF when exposed to NIR laser irradiation (Fig. 7B, C and **D**). After treatments, the tumor in each group was then stained with hematoxylin-eosin (H&E), ki67 and Terminal deoxynucleotidyl transferase-mediated dUTP nick-end labeling (TUNEL). The tumor cells in the NS and DTF groups were dense and complete in cell morphology with good overall growth status, demonstrating no obvious necrosis (Fig. 7E). Some nuclear crumpling and necrotic rupture were observed in DTF + NIR group, which was most likely because of the accelerated Fenton-like reaction mediated by NIR laser irradiation. The GA, GD, GDTF, and GDTF + NIR groups displayed distinct cell lysis and fragmentation as well as a substantial zone of necrosis. Necrosis was most pronounced in the GDTF + NIR group compared to the other groups, which might be due to the auto-amplified cancer therapy. There was almost no difference in ki67 staining between the NS and DTF groups (Fig. 7F). However, the yellow-brown cells in the DTF + NIR group was slightly lower. There were significantly fewer brownish yellow cells in GD, GDTF and GDTF + NIR groups than that in NS group, indicating that they had different abilities to suppress tumor growth. GDTF + NIR group had the lowest tumor cell growth, with very few yellow-brown cells visible, suggesting a significant inhibition of tumor cell proliferation. NS, DTF and DTF + NIR groups had closely packed tumor cells that expanded quickly, demonstrating almost little apoptosis (Fig. 7G). Some apoptotic cells were present in GA and GD groups, which was confirmed by weak green fluorescence. The strongest green fluorescence and the most apoptotic cells were found in GDTF + NIR group, demonstrating the best ability to induce cell apoptosis and the superiority of auto-amplified tumor therapy.

**Fig. 8:**
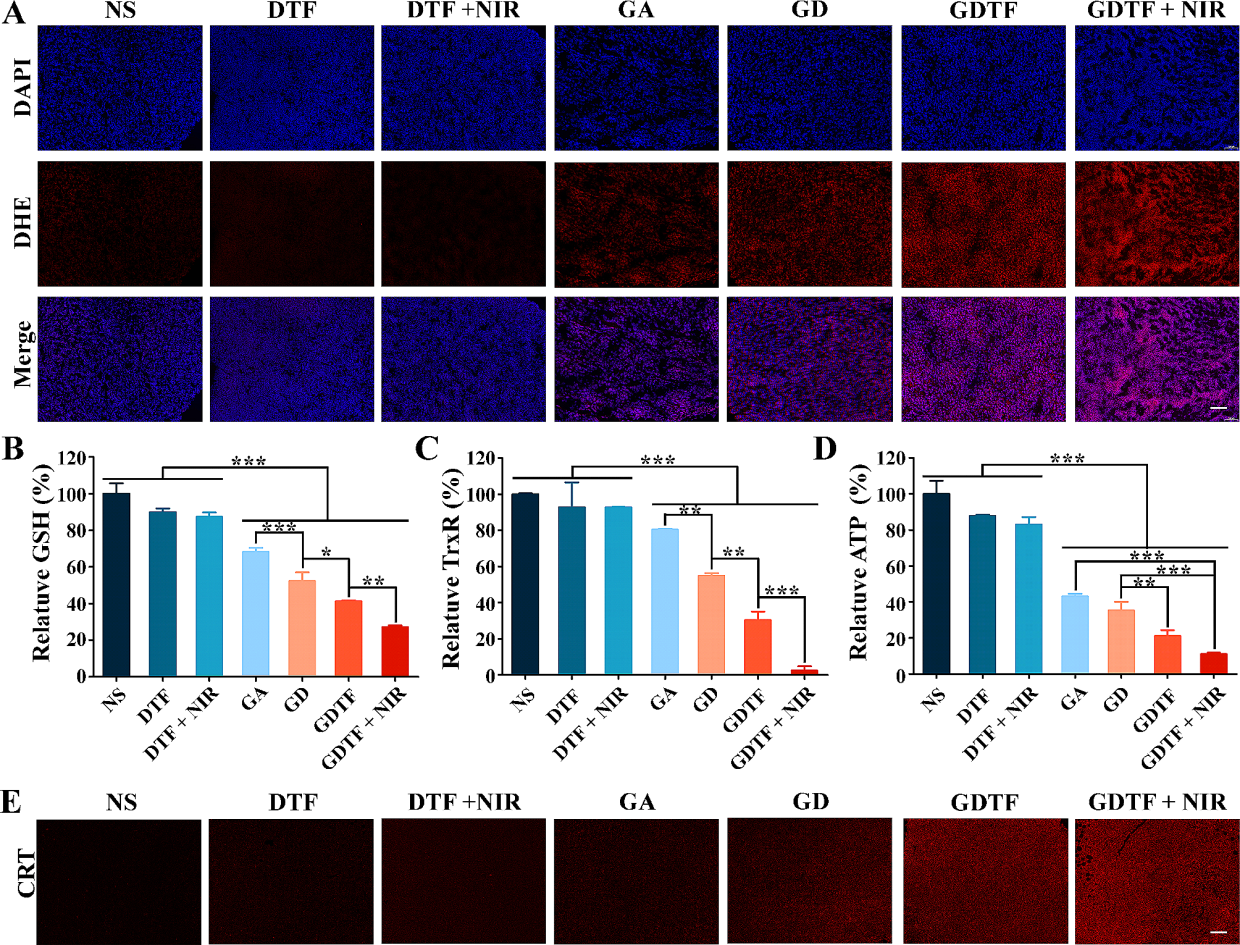
Antitumor mechanism of GDTF in vivo. (**A**) ROS generation of the tumors in each group detected using DHE probe. The scale bar is 100 μm. (**B**) Relative GSH content (%), (**C**) relative TrxR activity (%), and (**D**) relative ATP content (%) of the tumors in each group. ^*^P < 0.05, ^**^P < 0.01 and ^***^P < 0.001, significantly different. (**E**) CRT expression of the tumors in each group detected by immunofluorescence staining. The scale bar is 100 μm

The mechanism for the enhanced antitumor efficacy of GDTF in vivo was further determined. Tumor cells may be harmed under pathological circumstances because of the breakdown of the normal equilibrium between ROS generation and clearance. Therefore, the effect of GDTF on ROS generation in tumor tissues was further determined using DHE dye. The NS, DTF and DTF + NIR groups had almost invisible red fluorescence intensities, indicating almost no ROS generation (Fig. 8A). Different intensities of red fluorescence were present in the tumor tissues of GA, GD, GDTF and GDTF + NIR groups compared to the NS, DTF and DTF + NIR groups, demonstrating their various capacities to induce ROS generation. The highest level of ROS generation of GDTF + NIR group was confirmed by the presence of the strongest red fluorescence. This might be because of the mutual reinforcement of multiple effects, including increased tumor accumulation, enhanced pH/GSH dual-responsive drug release, GA-mediated chemotherapy, and improved Fenton-like reaction. Then, the causes of elevated ROS generation by GDTF were further examined by determination of GSH depletion and relative TrxR activity. Compared to NS group, DTF and DTF + NIR groups exhibited weak GSH depletion, but neither ROS generation nor effective tumor growth inhibition. This might be due to the GSH depletion being too weak to have an impact on ROS generation, collapse of the redox equilibrium, or efficient kill of cancer cells (Fig. 8B). However, they could assist in amplifying GA and GD to have a greater impact, leading to the improved anti-tumor efficacy of GD, GDTF, and GDTF + NIR groups. GD demonstrated a greater decreased relative TrxR activity compared to GA, which might be related to the increased tumor accumulation and GA release (Fig. 8C). GDTF further strengthened the ability to reduce relative TrxR activity, which might be due to the increased tumor accumulation by EPR and the hydrophilicity of TA. Additionally, the lowest relative TrxR activity of GDTF + NIR might describe to the enhanced tumor vascular permeability, cellular uptake, and drug release. As the primary energy molecule, ATP is crucial to many physiological and pathological cellular functions. Usually, a decline in ATP levels suggests diminished or compromised mitochondrial function. Inspired by the reduction in MMP and ATP caused by GDTF in vitro, the ATP level was further determined in vivo. Compared with NS, DTF and DTF + NIR slightly reduced ATP (Fig. 8D). GA strongly reduced ATP, however, its exact mechanism remained unknown and required further study. GD, GDTF and GDTF + NIR groups gradually decreased ATP levels, which might be due to the synergistic effects of the elevated tumor accumulation and cellular uptake, NIR-enhanced pH/GSH dual-responsive drug release, and NIR-improved Fenton-like reaction. In conclusion, GDTF + NIR might increase intracellular ROS levels to disrupt mitochondrial function through GSH depletion and reduce relative TrxR activity, which in turn caused a loss in ATP generation, cellular insufficiency, and ultimately tumor apoptosis or death. Moreover, GDTF and GDTF + NIR displayed apparent red fluorescence, demonstrating calreticulin (CRT) exposure (Fig. 8E). This indicated that ROS generation induced by GDTF + NIR might induce immunogenic cell death (ICD) and provoked anti-tumor immune response to improve anti-tumor efficacy. It has been reported that a transient intracellular ROS surge could stimulate dendritic cells (DCs) maturation to initiate adaptive T cell responses [50]. The specific action mechanism of immune response induced by GDTF + NIR need to be studied.

Finally, the biocompatibility of GDTF was determined. The body weight change curves of the treatment groups (DTF, DTF + NIR, GA, GD, GDTF, and GDTF + NIR groups) did not differ substantially from that of NS group (Figure S20A). The results of H&E staining of major organs in treatment groups, including heart, liver, spleen, lung, and kidneys, were not significantly different from those of the NS group, confirming that GDTF did not cause significant damage to major organs (Figure S21). There were no discernible differences between the treatment groups and NS group in the indices related to erythrocytes (HCT, RBC, MCV, MCHC, MCH, RBC), leukocytes (WBC, LY#, MO#, LY%, MO%), platelets (PLT, MPV, PCT, PDW, PLCR), and hemoglobin (HGB, MCHC, MCH) (Figure S20B-D). Additionally, none of the groups displayed any glaring AST, ALT, CREA, or UREA abnormalities, demonstrating that GDTF did not cause discernible liver or kidney damage (Figure S22). In conclusion, GDTF demonstrated good biocompatibility.

## Conclusion

GDTF was successfully constructed by wrapping GA-loaded DPSN with a TA-Fe(III) coating layer and demonstrated enhanced antitumor efficacy with good biocompatibility both in vitro and in vivo. GDTF enhanced the tumor accumulation and cellular uptake by EPR effect of GDTF, hydrophilicity and cell adhesion of TA. GDTF + NIR was endowed with the property of NIR-enhanced pH/GSH dual-responsive drug release due to the features of photothermal conversion, pH-responsive degradation of TA-Fe(III) layer, and GSH-responsive degradation of DPSN. GDTF + NIR demonstrated the capacity to elevate intracellular ROS, deplete GSH, and reduce TrxR activity, resulting in decreased ATP, cellular insufficiency, and eventually cell apoptosis or death. This research provided great potential for auto-amplified cancer therapy based on disrupting redox equilibrium.

## Materials and methods

### Materials

Cetyltrimethylammonium chloride (CTAC), bis[3- (triethoxymethylsilyl) propyl]tetrasulfide (BTES), fluorescamine, tannic acid (TA), glutathione (GSH), methylene blue (MB), Triton X-100 and Cyanine 3 (Cy3) were purchased from Shanghai Aladdin Biochemical Technology Co., Ltd. Triethanolamine (TEA) was obtained from Tianjin Tianli Chemical Reagents Co., Ltd. Tetraethyl orthosilicate (TEOS) was purchased from Shanghai Macklin Biochemical Technology Co., Ltd. (3-Aminopropyl)triethoxysilane (APTES) was obtained from Shanghai Meiyuan Biochemical Technology Co., Ltd. Gambogic acid (GA) was purchased from Chengdu Push Bio-technology Co., Ltd. Ferric chloride (FeCl_3_) was obtained from Tianjin Zhiyuan Chemical Reagent Co. Ltd. Fluorescein 5-isothiocyanate (FITC) was purchased from Sigma-Aldrich. 3-morpholinepropanesulfonic acid (MOPS) and the thioredoxin reductase (TrxR) enzyme activity assay kit were purchased from Beijing Solarbio Science & Technology Co. Ltd. 5,5’-Dithiobis-(2-nitrobenzoic acid) (DTNB) was obtained from Shanghai Haohong Biomedical Technology Co. Ltd. Cell counting kit-8 (CCK-8) cell proliferation and cytotoxicity assay kit was obtained from Dalian Meilun Biotech. Co. Ltd. Calcein-AM/PI, live/dead cell double staining kit was purchased from Beijing Biotopped Life Sciences Co. Ltd. Reactive oxygen species assay kit, mitochondrial membrane potential assay kit with JC-1, GSH and GSSG assay kit, and enhanced ATP assay kit were purchased from Beyotime Biotech. Inc.

### Preparation of DPSN

DPSN was prepared according to the method reported previously [25, 35]. CTAC (0.5 g) and TEA (0.06 g) were added to 20 mL water and mixed. Thereafter, the mixture was stirred at 80 ℃ for 20 min, resulting in solution (A) A certain amount of TEOS and 0.2 mL of BTES were mixed, and the mixture was ultrasonicated for 30 min to form solution (B) The solution B was slowly added dropwise to the solution A and the stirring was continued at 80 ℃ for 4 h. The precipitate was collected by centrifugation (15,000 rpm, 25 min) and rinsed with anhydrous ethanol. To remove CTAC, the resulting white precipitate was dispersed in 20 mL of template remover solution, and the solution was stirred or alternatively refluxed for 12 h. The precipitate was collected by centrifugation (10,000 rpm, 10 min) and washed with anhydrous ethanol. This procedure of template removal was performed for multiple times. Finally, the precipitate was collected and dried under vacuum at 60 °C to obtain DPSNs. To find out the optimal synthesis technique, TEOS volume, template remover, and times of stirring during template removal were investigated.

### Preparation of GD

DPSNs was first modified with amino (-NH_2_) by conjugating with APTES. The synthesized DPSNs (5 mg) were resuspended in 10 mL water and then mixed with 0.1 mL APTES while stirring for 8 h. The content of -NH_2_ on the surface of DPSNs-NH_2_ was determined by fluorescence spectrophotometry as reported [51]. DPSNs-NH_2_ and GA were dissolved in 10 mL methanol with various mass ratios of GA to DPSN-NH_2_, following stirring at room temperature for 24 h. The precipitate was collected following centrifugation and rinsed three times. The precipitate was dried under vacuum to obtain GD.

### Preparation of GDTF

10 mg GD were ultrasonically dispersed in water before being combined with TA solutio. After stirring for 5 min, the afore-mentioned solution was mixed with FeCl_3_·6H_2_O in MOPS buffer solution and stirred at room temperature. GDTF were achieved by centrifugation and washing with water for 3 times.

### Characterization of nanoparticles

The prepared DPSN, GD and GDTF nanoparticles were dissolved in water in a Celine bottle, and their appearance and dispersion were observed and photographed. The appearance morphology, hydrodynamic size, zeta potential were investigated by transmission electron microscope (TEM, G2 F30 S-TWIN, FEI, US) and Nano ZS Zetasizer (Zetasizer Nano ZS-90, Malvern, UK), respectively. Infrared spectra and differential scanning calorimetry (DSC) spectra of the prepared DPSN, GD and GDTF were tested by Fourier transform infrared spectrometer (FTIR, Nicolet iS50, Thermo Fisher Scientific, US) and X-ray photoelectron spectroscopy (XPS, ESCALAB QXi XPS, Thermo Fisher Scientific, US) respectively. The Fe content in GDTF was measured by Inductively Coupled Plasma-Optical Emission Spectrometer (ICP-OES, Agilent 730, Agilent, US).

### Drug loading and encapsulation efficacy

The drug loading (DL, %) and encapsulation efficacy (EE, %) of GA in GDTF were determined by UV spectrophotometry (λ_max_ = 361 nm). The DL and EE values were then calculated using the following equations: DL (%) = (W_1_-W_2_)/W_3_ × 100; EE (%) = (W_1_-W_2_)/W_1_ × 100. Here, W_1_, W_2_, and W_3_ correspond to the total weight of GA, and the mass of free GA, and the total weight of GDTF.

### Photothermal conversion capacity

GDTF solutions at various concentrations (0, 25, 50, 100, 200 and 300 µg/mL) were exposed to a 808 nm laser irradiation at the power density of 2.5 W/cm^2^ for 600 s. The temperature changes were recorded by an infrared thermal imaging camera (Fluke Ti200, Fluke, US) to investigate the effects of GDTF concentration, irradiation time and power density on the photothermal conversion efficacy. To investigate the photothermal conversion stability of GDTF solution, five ON/OFF irradiation cycles experiment was performed.

### Drug release

2 mL GDTF solutions were placed into a dialysis bag (MWCO, 3500 Da) and then immersed in tubes containing 10 mM GSH or GSH-free phosphate buffer solution (PBS) at different pH values (7.4, 6.5, 5.0), respectively. They were placed in a constant temperature shaker at 37 ℃ while stirring at 100 rpm. To evaluate the effect of laser irradiation on drug release, the samples were exposed to a 808 nm laser at a power density of 2.5 W/cm^2^ for 10 min. At different time points (0.5, 1, 2, 4, 6, 8, 10, 12, 24 h), 1 mL sample solution was removed and immediately replaced with an equal volume of freshly warmed buffer. The concentration of GA in each medium was quantified by UV spectrophotometry and the cumulative percent drug release (%) was calculated according to the standard curves.

### GSH depletion

DTNB was utilized as an indicator to assess the GSH depletion of GDTF, as DTNB reacts with the sulfhydryl group on GSH to form yellow 2-nitro-5-thiobenzoic acid (TNB). 2 mL GSH solutions at various concentrations (0, 20, 40, 60, 80 and 100 µM) were mixed with 50 µL DTNB (10 mM). The absorption spectra were measured in the range of 400–600 nm and the GSH standard curve was plotted according to the absorbance at 412 nm. DPSN, GA, GD, GDTF were respectively added to a buffer solution containing GSH (1 mM, pH 5.0), which were incubated at 37 ℃ while stirring at 100 rpm for 2 h. H_2_O_2_ (10 mM) and water were employed as positive and negative control, respectively. The supernatant was collected following centrifugation and mixed with 50 µL of 10 mM DTNB for measuring the absorbance at 412 nm. The effects of pH and laser irradiation on GSH depletion were determined.

### Determination of hydroxyl radicals (•OH) production

MB degradation experiments were performed to determine the ability of GDTF to generate hydroxyl radicals (•OH) [52, 53]. GDTF, MB, H_2_O_2_ and GSH solution were mixed well together. The samples were incubated in a constant temperature shaker at 37 ℃, 100 rpm, and in the dark for 2 h. The supernatant was collected following centrifugation and the MB content was quantified by the absorbance at 665 nm. Similarly, the influences of pH, GSH, laser irradiation and ATP on •OH production were determined.

### Stability

Particle size and EE% were applied as indicators to investigate the stability of GDTF placed at room temperature for 30 days.

### Hemolysis assay

The hemolysis rate assay was performed to evaluate the erythrocyte compatibility of GDTF as reported before [54, 55]. GDTF at different concentrations were combined with 2% erythrocyte suspension respectively, and then placed in a constant temperature water bath at 37 °C while shaking for 4 h. The supernatant was collected following centrifugation and detected at 540 nm. PBS and Triton X-100 were applied as the negative control and positive control, respectively.

### Cellular uptake

Both fluorescence microscope and UV-Vis spectrophotometry were used to evaluate the cellular uptake of GDTF. To observe the cellular uptake behavior of GDTF in 4T1 cells using a fluorescence microscopy, CFDTF was prepared by swapping FITC-labeled APTES and Cy3 for APTES and GA, respectively. 4T1 cells were seeded into a 12 well-plate at a density of 1.0 × 10^5^ cell/well and the cells were incubated. Each well was rinsed three times with PBS and then cultured with fresh complete cultivation containing FITC, Cy3, CFD, and CFDTF, respectively. To investigate the impact of NIR irradiation on the cellular uptake, 4T1 cells were incubated with CFDTF for 4 h before being exposed to 808 nm laser irradiation for 10 min at a power density of 2.5 W/cm^2^. For visualized observation, the nucleus of 4T1 cells were stained with DAPI for 10 min. After the cells were washed, they were observed using a fluorescence microscope (CKX35, Olympus, Japan). For quantitative analysis, 4T1 cells were seeded into a 6 well-plate at a density of 3.0 × 10^5^ cell/well and incubated with GA, GD and GDTF for various time points. Then, the cell culture media were collected and mixed with methanol at a volume ratio of 1:1, followed by centrifugation. The content of GA in the collected supernatant suspension was determined by UV-Vis spectrophotometry.

### Cytotoxicity

4T1 cells were seeded on 96 well-plates at a density of 8 × 10^3^ cells per well before being incubated in a 37 °C, 5% CO_2_ cell culture incubator. Once the cells achieved a certain density, the original media was discarded and replaced with fresh medium containing DPSN, DTF, GA, GD and GDTF at various concentrations. Each well was incubated for 48 h and incubated with 10 µL CCK-8 solution for 4 h. To investigate the impact of NIR irradiation on cell cytotoxicity, the cells were incubated with DTF and GDTF for 4 h before being exposed to 808 nm laser irradiation for 10 min at a power density of 2.5 W/cm^2^, and then incubated for 44 h.A microplate reader (Multiskan Sky 1510, Thermo Scientific, US) was used to detect the absorbance at 450 nm, and the formula was used to calculate and plot the cell viability curve.

### Live/dead staining

4T1 cells were seeded into a 12-well plate, and then incubated for 24 h. Each well was rinsed three times with PBS and then cultured with fresh complete cultivation containing PBS, DPSN, DTF, GA, GD, and GDTF respectively, for 24 h. To investigate the impact of NIR irradiation on the cell apoptosis, 4T1 cells incubated with DTF and GDTF for 4 h were exposed to 808 nm laser irradiation for 10 min at a power density of 2.5 W/cm^2^, and then incubated for 20 h. Each well was rinsed with PBS for 3 times and stained with Calcein-AM and PI in the dark for 30 min. The cells were washed three times with PBS, and then observed using a fluorescence microscope.

### Determination of intracellular ROS

A 6-well plate was seeded with 4T1 cells at the density of 3.0 × 10^5^ cells per well and cultured for 24 h. After being rinsed three times with PBS, each well was incubated with fresh cell medium containing PBS, DPSN, DTF, GA, GD, and GDTF for 24 h. After being incubated with DTF and GDTF for 4 h, the cells were then exposed to an 808 nm laser for 10 min at a power density of 2.5 W/cm^2^ to determine the effect of laser irradiation on intracellular ROS generation. Each well was washed three times with PBS and stained with DCFH-DA in the dark for 30 min. Thereafter, intracellular ROS generation was determined using a fluorescence microscopy.

### Determination of MMP

The procedure for cell seeding, culturing, and dosing was the same as that described in the “*Determination of intracellular ROS*” section. Then, followed the guidelines provided with the commercial MMP assay kit with JC-1. Specifically, the cells were incubated with JC-1 staining working solution at 37 °C for 20 min. After washing with JC-1 staining buffer, the cells were cultured with 1 mL cell culture media and observed using a fluorescence microscope.

### Determination of GSH depletion in vitro

The procedure for cell seeding, culturing, and dosing was the same as that described in the “*Determination of intracellular ROS*” section. Then, follow the guidelines provided with the commercially GSH and GSSG assay kit. After incubation, the cells were rinsed with PBS and collected by a centrifugation. The cell pellet was added to the protein removal reagent M solution in a volume of 3 times and vortexed vigorously. The samples were then subjected to two times of rapid freeze-thaw using liquid nitrogen and a 37 ℃ water bath. The samples were incubated at 4 °C for 10 min, centrifuged at 10, 000 g for 10 min, and the supernatant was removed, kept on ice, for total GSH analysis. The sample solutions, protein removal reagent M solution, and working solution for the total GSH assay were mixed to prepare the reactions in 96-well plates. After fully blending, the mixture in each well was incubated for 5 min at room temperature. Next, 50 µL of NADPH solution at 0.5 mg/mL was added into the mixture in each well. Based on the microplate reader-measured absorbance at 412 nm, the GSH concentration was calculated.

### Determination of intracellular TrxR activity

The effect of GDTF on TrxR activity in 4T1 cells was detected using a thioredoxin reductase (TrxR) activity assay kit. The procedure for cell seeding, culturing, and dosing was the same as that described in the “*Determination of intracellular ROS*” section. Following incubation, the cells were collected, twice-washed with PBS, and broken into smaller pieces by ultrasonic waves in an ice bath in a ratio of the number of cells (1.0 × 10^4^): the volume of reagent one (mL) of 500–1000:1. Then, the cells were centrifuged at 10, 000 rpm and 4°C for 10 min. The supernatant was removed and placed on ice for analysis. For each well, the TrxR activity was determined by measuring the absorbance at 412 nm using a microplate reader.

### Determination of intracellular ATP

The effect of GDTF on intracellular ATP in 4T1 cells was detected using an enhanced ATP assay kit. The procedure for cell seeding, culturing, and dosing was the same as that described in the “*Determination of intracellular ROS*” section. After incubation, the culture media in each well was discarded and replaced with 200 µL lysis buffer to lyse the cells. The cells were completely lysed by pipetting the lysis buffer up and down several times, and then centrifuged at 12, 000 g for 5 min at 4 °C, with the supernatant being collected for the subsequent experiments. Then, 20 µL of the sample or standard was added to each assay well, immediately mixed by pipetting, and determined using a microplate reader.

### Animal studies

Female specific-pathogen-free (SPF) BALB/c mice (6–8 weeks old, 21 ± 2 g) were purchased from Ji’nan Pengyue Laboratory Animal Breeding Co., Ltd. (Shandong, China). They acclimatized under 12 h/12 h light/dark cycles at a constant temperature (23 ± 2 ℃), humidity 50-60% and were provided with tap water and a pelleted basal diet before the start of the experiments. All animal experiments were approved by the Committee on the Ethics of Animal Experiments of Henan University of Chinese Medicine (Henan, China).

### Construction of the subcutaneous 4T1 tumor model

BALB/c mice were subcutaneously injected with 0.1 mL of 4T1 cells at the concentration of 1.0 × 10^7^ cells/mL with good growth status. The mice were then placed in an SPF-grade environment for rearing, fed and watered ad libitum, and the growth of the tumors was regularly monitored. Vernier caliper was used to measure the long diameter (D) and short diameter (d) of tumor, and the tumor volume was determined using the formula V = 0.5 × D × d^2^.

### Biodistribution in vivo

The small molecule medication GA was substituted with IR780 for preparation of ID and IDTF. The abovementioned 4T1 tumor-bearing mice were randomly divided into three groups and injected via tail vein with 100 µL IR780, ID and IDTF (IR780 at 2.0 mg/kg) respectively. After injection for 2, 4, 8, 12, 24 and 48 h, the biodistribution of IR780 was imaged using an In-Vivo Xtreme Imaging System (Bruker, US). The mice were sacrificed and the major organs including heart, liver, spleen, lung, kidney, and tumor were collected at 48 h post-injection for investigating the biodistribution of IDTF.

### Tumor growth inhibition

The abovementioned 4T1 tumor-bearing mice were randomly divided into seven groups: normal saline (NS, control), DTF, DTF + NIR, GA, GD, GDTF, and GDTF + NIR (*n* = 6). The mice in each group were administered with 100 µL the corresponding drug solution at GA of 2.0 mg/kg once every three days for a total of five times. The mice in groups of DTF + NIR and GDTF + NIR were exposed to an 808 nm laser at the power density of 1.5 W/cm^2^ for 5 min at 4 h post-injection. The tumor volume of each group was measured using a vernier caliper prior to each administration, and the tumor growth curves in the tumor-bearing mice were plotted throughout the treatment. After treatments, the mice were sacrificed and the tumors were collected, photographed, weighed, and calculated for tumor inhibition rate (%).

### H&E, ki67 and TUNEL staining

The tumor tissues were obtained, fixed in 10% formalin solution, dehydrated, embedded in paraffin, or stored at -80 °C for 2 h, sliced or cryosectioned into 5 m sections, stained with H&E (Servicebio, cat. no. G1031) and TUNEL assay kit (Servicebio, cat. no. GDP1042) following routine protocols respectively, and then were observed on a microscope (Olympus BX-41/Q-Color3, Japan). Tumors from the sacrificed mice in each group were immersed in 10% formalin solution, embedded in paraffin, and subjected to be assessed by immunohistochemical (IHC) staining. Then, tumors were cut into slices using a cryostat, which were mounted on glass slides, and stained with anti-Ki67 Mouse mAb (Servicebio, cat. no. GB121141-100).

### Determination of ROS in vivo

The tumor samples from each group were collected, OCT-embedded, snap-frozen in liquid nitrogen, and cryo-sectioned. After being stained with DHE for 0.5 h, ROS in tumors from each group was observed using an inverted fluorescence microscope.

### Determination of GSH, TrxR and ATP in vivo

The tumors collected from each group were grinded into powder by snap-freezing with liquid nitrogen. For every 10 mg of powder, 30 µL of Protein Removal Reagent M solution was added and vortex rapidly, and then 70 µL of Protein Removal Reagent M solution was added to thoroughly homogenize with a glass homogenizer. After an incubation at 4 ℃ for 10 min, the samples were centrifuged at 10,000 g for 10 min at 4 ℃. The supernatant was collected and kept on ice or at 4 ℃ for total GSH analysis. The GSH concentration in tumor was determined and GSH depletion was calculated according the assay procedures described in the section of “GSH depletion in vitro”.

For every 10 mg of tumor, 100 µL of reagent I was added and then homogenized using a glass homogenizer. After an adequate homogenization, the samples were centrifuged at 10,000 rpm and 4 ℃ for 10 min and the supernatant was collected for subsequent assay. The TrxR activity in tumors was determined according the assay procedures described in the section of “Determination of intracellular TrxR activity”.

For every 10 mg of tumor, 200 µL of Lysis Buffer was added and then homogenized using a glass homogenizer. After an adequate homogenization, the samples were centrifuged at 12,000 g and 4 ℃ for 5 min and the supernatant was collected for subsequent assay. The ATP concentration in tumor was determined according the assay procedures described in the section of “Determination of intracellular ATP”.

### Biocompatibility in vivo

The body weight of each group was measured using an electronic balance prior to each administration, and their body weight change curves were plotted throughout the treatment. The collected major organs including heart, liver, spleen, lung, and kidney were stained with H&E (Servicebio, cat. no. G1031) and histologically examined. The harvested blood samples from each group were centrifuged at 3,000 g and 4 °C for 15 min, aliquoted and kept at 80 °C for the subsequent assay. An automatic biochemical analyzer (Chemray 800, Rayto, China) was used to measure the function of the liver and kidneys. After anticoagulant was administered to the whole blood, and an automatic animal blood cell analyzer (BC-2800 Vet, Mindray Animal Care, China) was used to measure hematological indicators.

### Statistical analysis

In this study, all data were presented as mean ± standard deviation (SD). Student’s t-test analysis or one-way ANOVA was applied. Bonferroni’s post hoc test was used the data. Repeated measures analysis of variance was used for repeated measures data. Nonparametric tests were utilized for data that were not normally distributed. A P-value of < 0.05 was considered statistically significant. Statistical analysis was performed using GraphPad Prism software (GraphPad Prism, version 5).

### Electronic supplementary material

Below is the link to the electronic supplementary material.

Supplementary Material 1

## Data Availability

No datasets were generated or analysed during the current study.
