# Supplementary material for: pH/GSH dual-responsive nanoparticle for auto-amplified tumor therapy of breast cancer
Source: J Nanobiotechnology. 2024 Jun 10;22:324. doi: 10.1186/s12951-024-02588-0 (PMC11163783; doi:10.1186/s12951-024-02588-0)
Supplement: Supplementary file 1 — Supplementary Material 1 [file 12951_2024_2588_MOESM1_ESM.doc]

Supplementary Material

**pH/GSH Dual-responsive Nanoparticle for Auto-amplified Tumor Therapy of Breast Cancer**

*Shengnan Huang^1, 2*^,* *Zhiling Xu^3^, Weiwei Zhi^3^, Yijing Li^3^, Yurong Hu^2^, Fengqin Zhao^2^, Xiali Zhu^3*^, Mingsan Miao^1*^, Yongyan Jia^3*^*

**
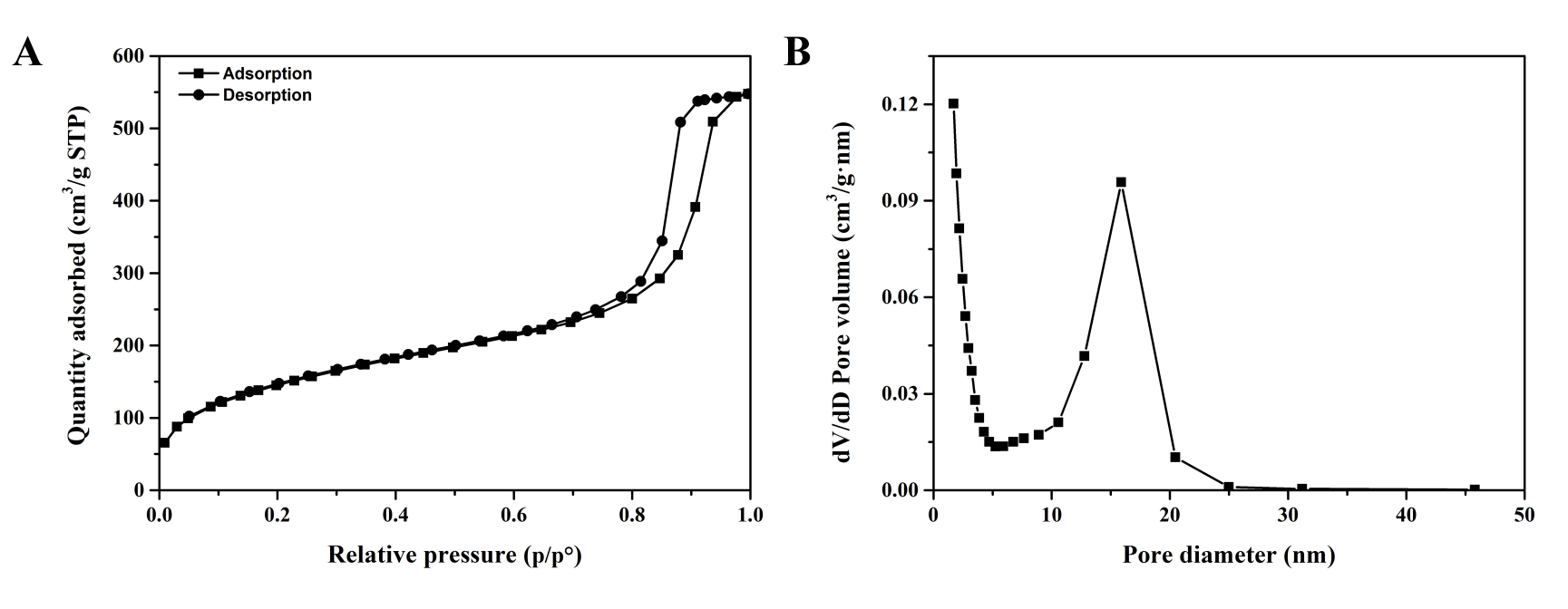
**

**Figure S1.** (A) N_2_ absorption-desorption curve and (B) pore diameter distribution curve of DPSN.


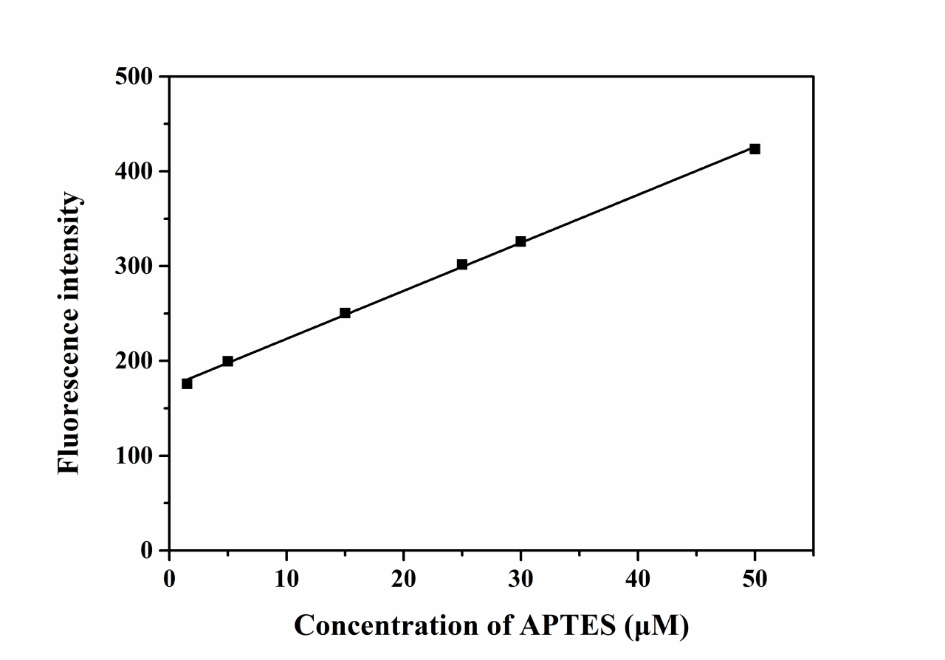


**Figure S2.** Standard curve for the determination amine content of APTES.


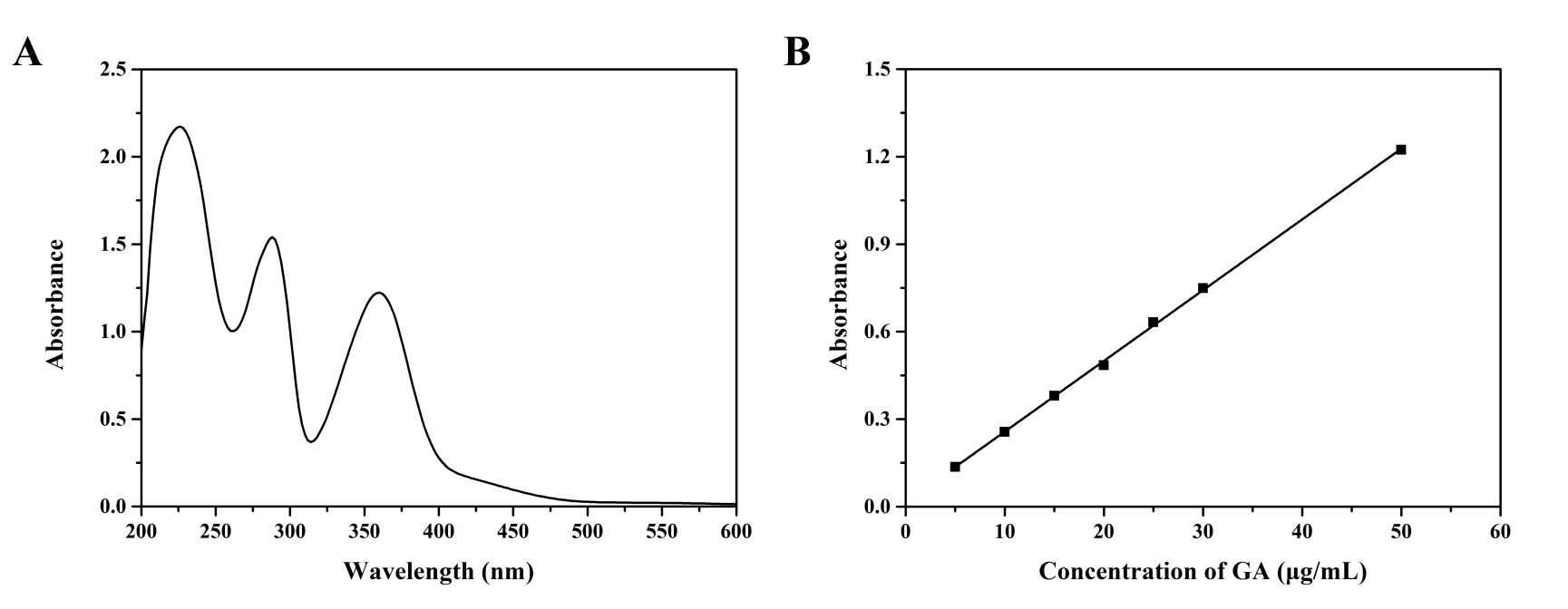


**Figure S3.** (A) UV absorption spectrum of GA. (B) Standard curve of GA.


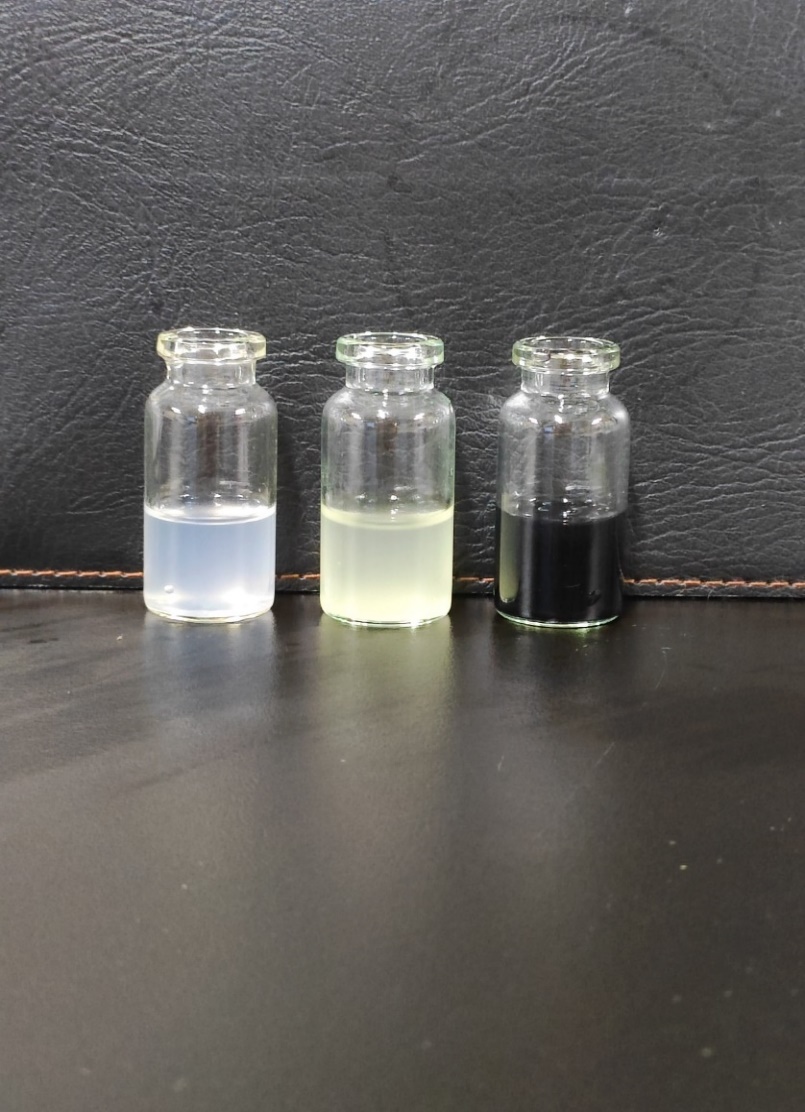


**Figure S4**. Photographs of DPSN (left), GD (middle) and GDTF (right).


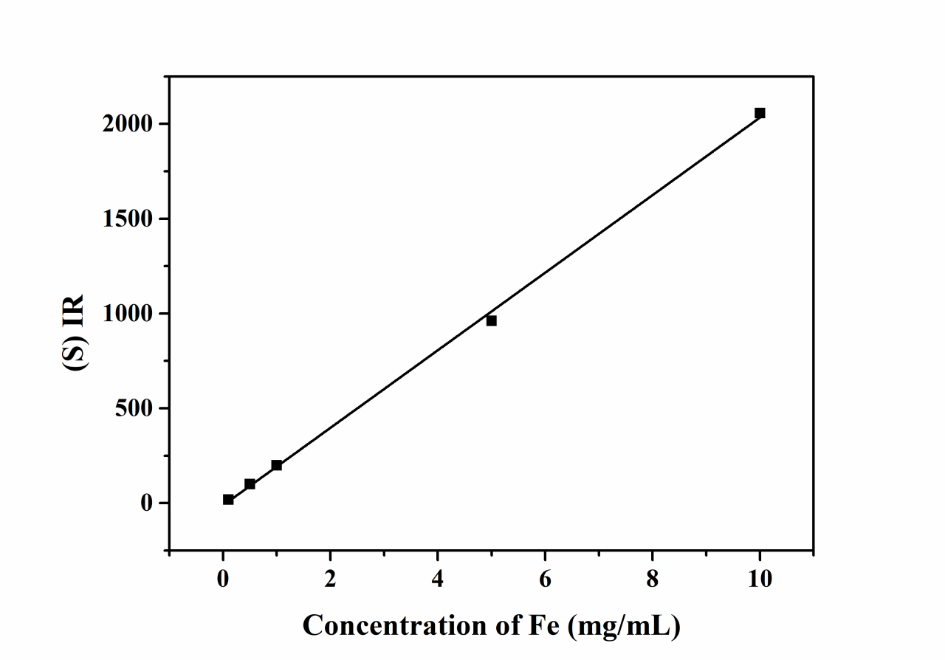


**Figure S5.** Standard curve of Fe determined by ICP-OES.


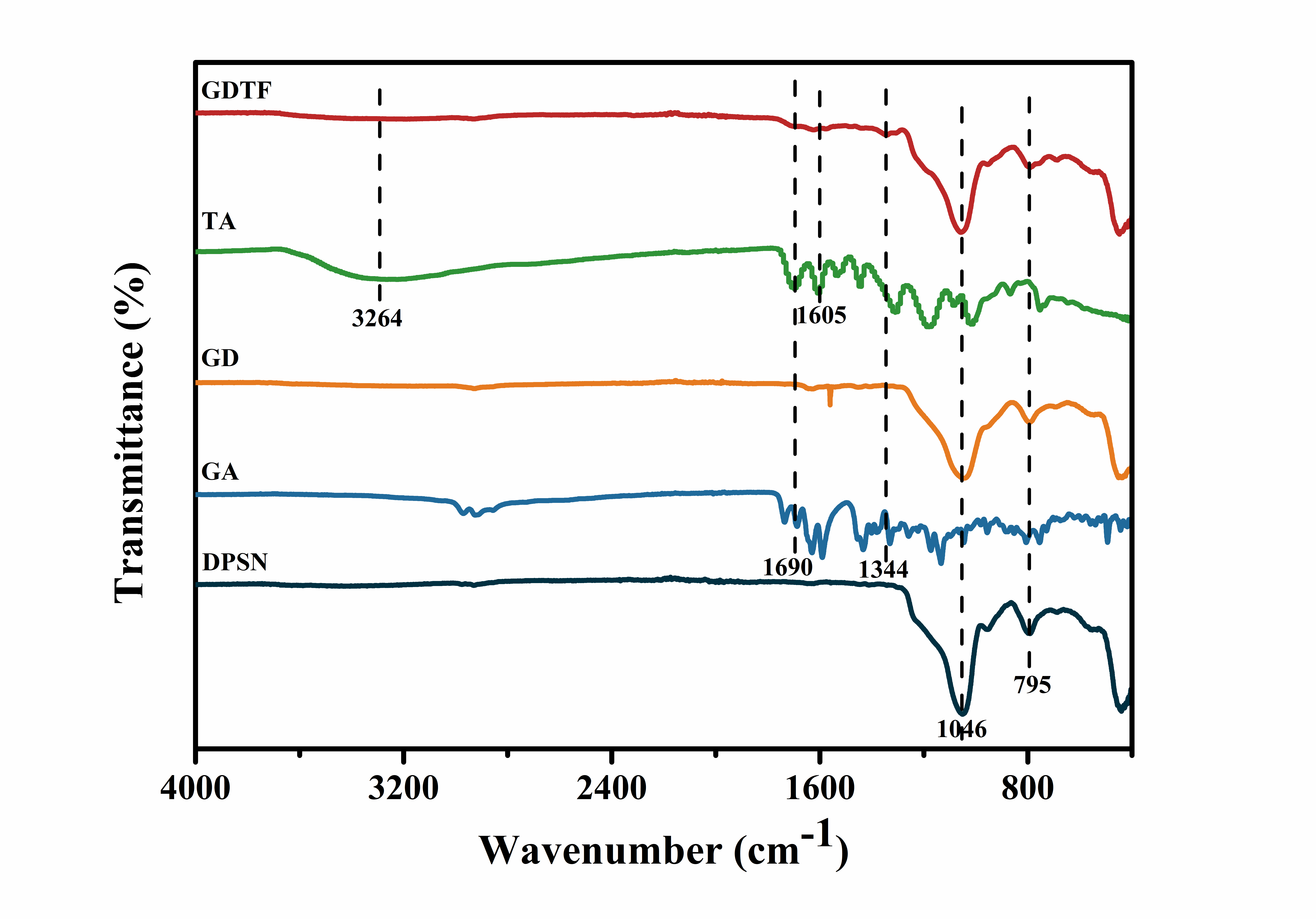


**Figure S6.** FT-IR spectra of DPSN, GA, GD, TA and GDTF.


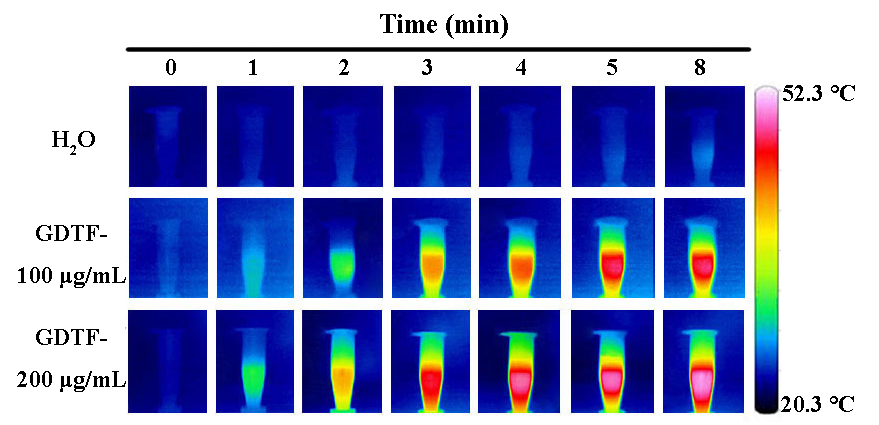


**Figure S7.** Infrared thermograms of GDTF at various concentrations during exposure to 808 nm laser irradiation.


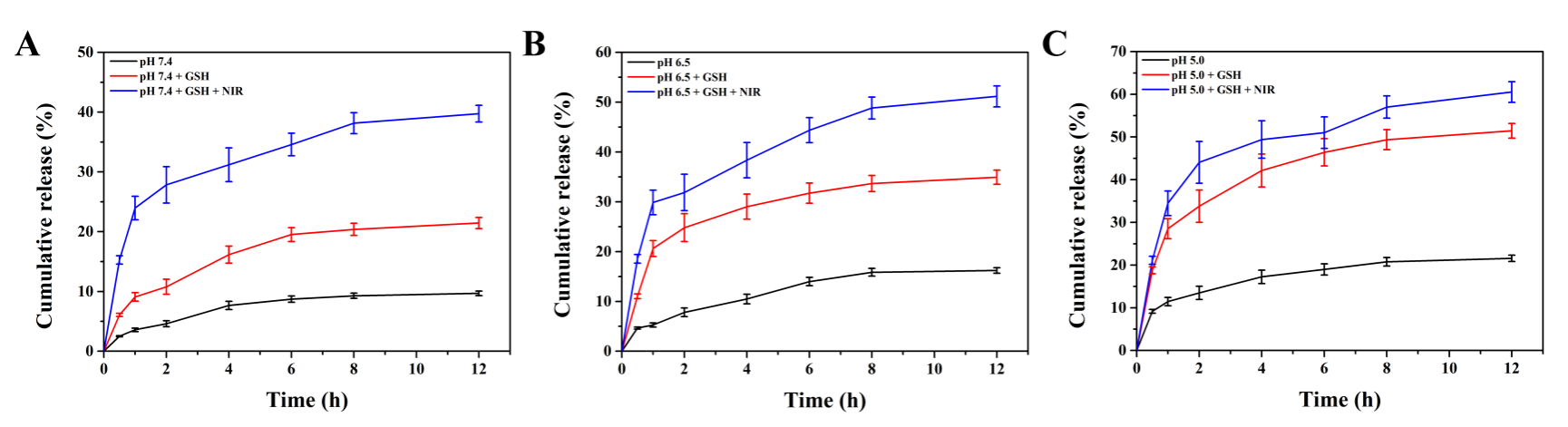


**Figure S8.** GA release from GDTF under different conditions.


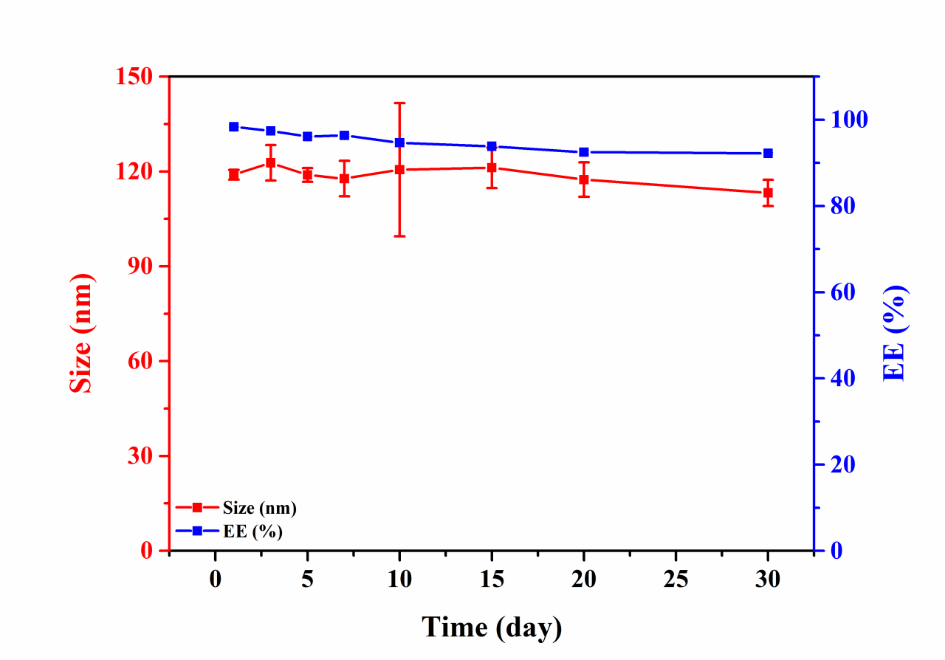


**Figure S9.** Changes in hydrodynamic size and EE% of GDTF during 30 days at room temperature.


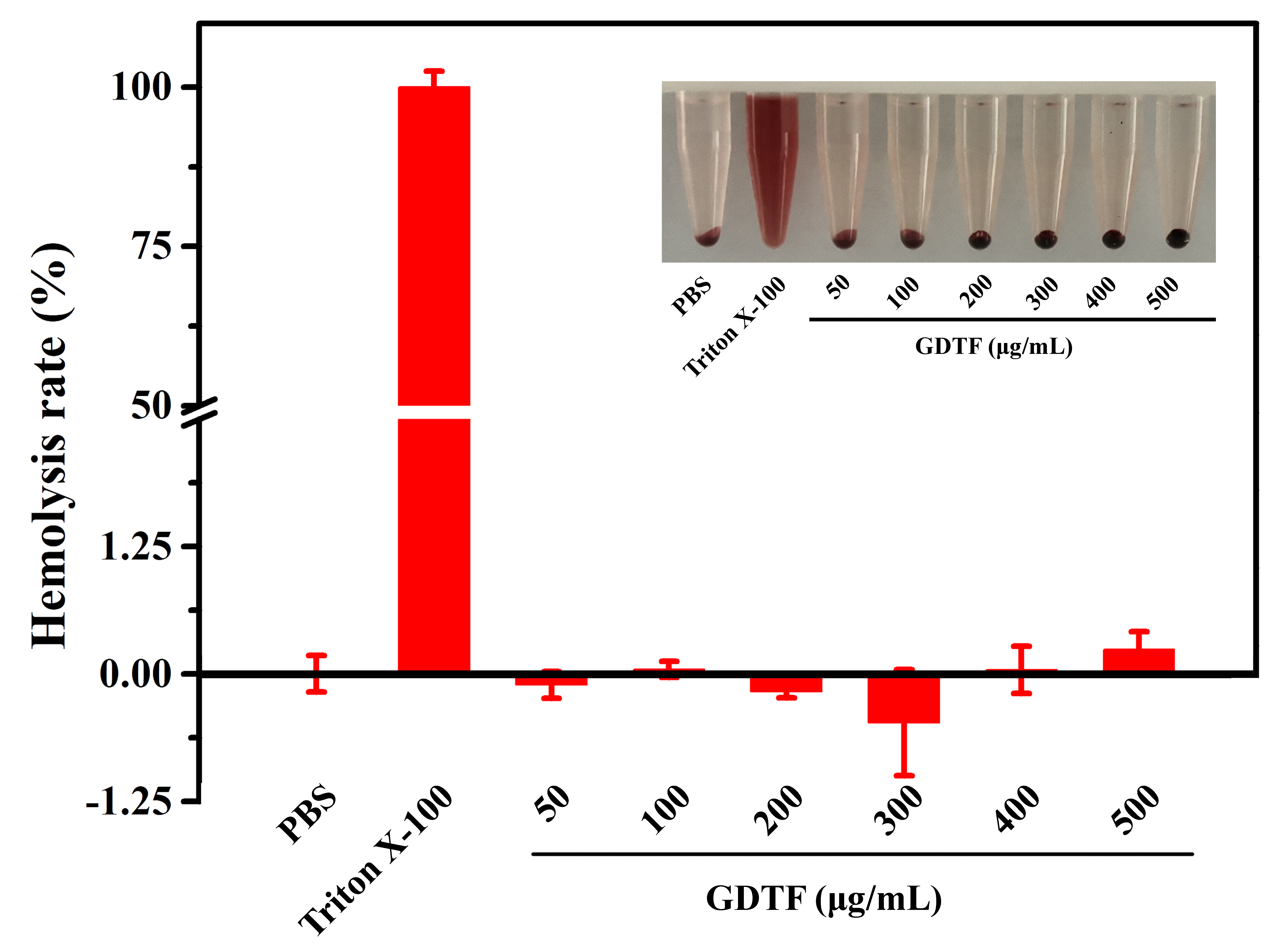


**Figure S10.** Hemolysis rate of PBS (negative control), Triton X-100 (positive control) and GDTF at various concentrations.


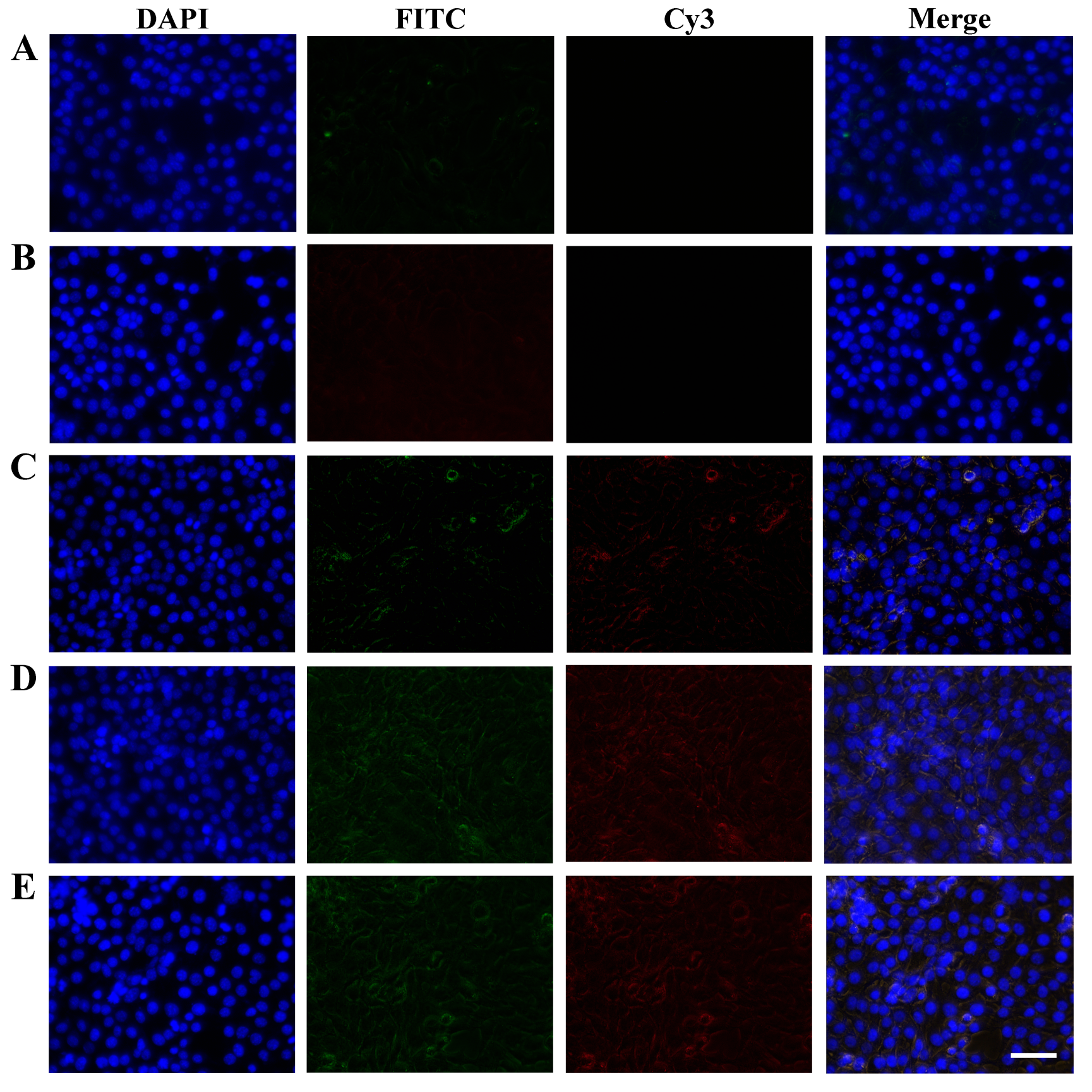


**Figure S11.** Cellular uptake of (A) FITC, (B) Cy3, (C) CFD, (D) CFDTF, (E) CFDTF + NIR in 4T1 cells for 1 h. For group E, the cells were incubated with CFDTF for 1 h and then exposed to 808 nm laser irradiation at the power density of 2.5 W/cm^2^ for 10 min. Scale bar is 50 μm.


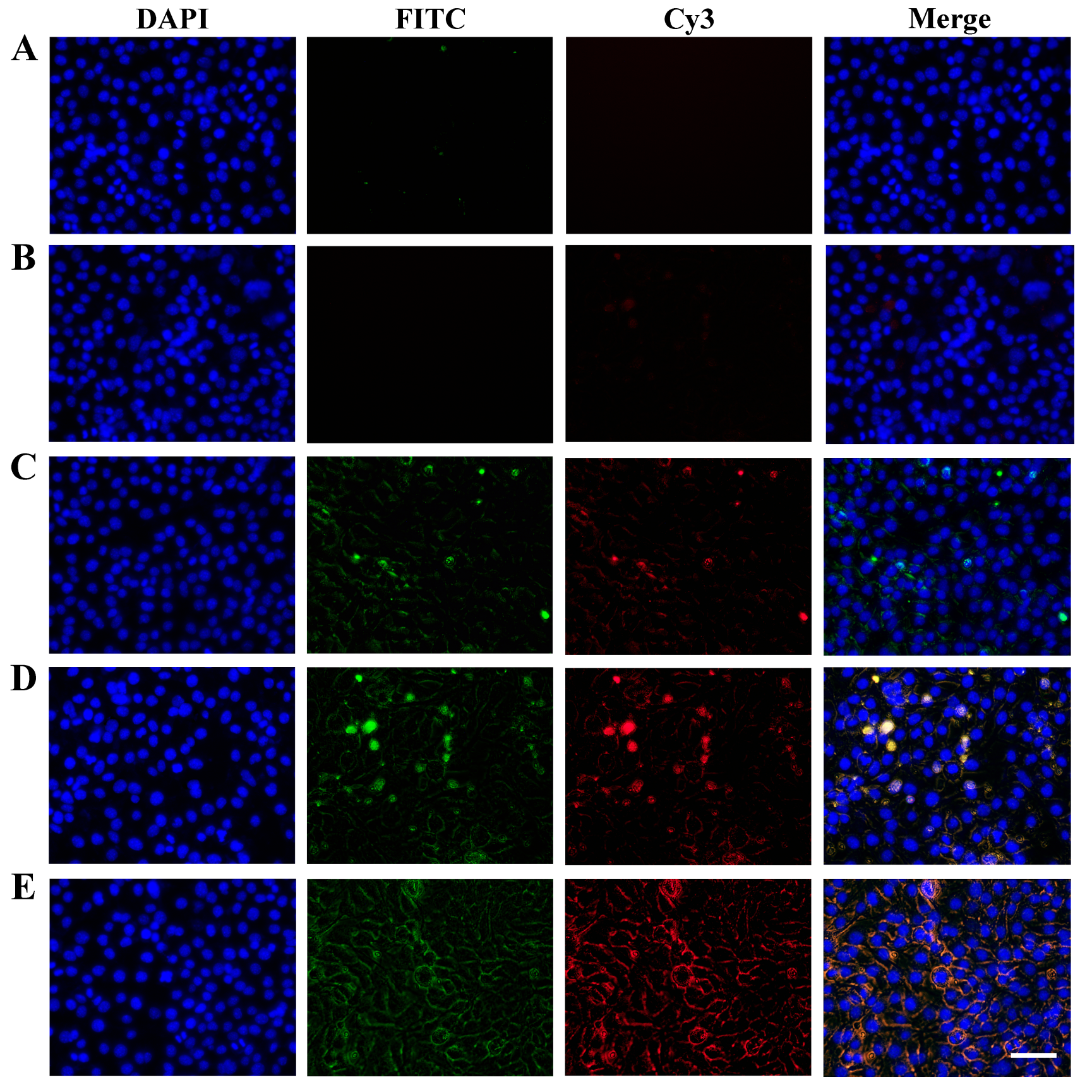


Figure S12. Cellular uptake of (A) FITC, (B) Cy3, (C) CFD, (D) CFDTF, (E) CFDTF + NIR in 4T1 cells for 2 h. For group E, the cells were incubated with CFDTF for 2 h and then exposed to 808 nm laser irradiation at the power density of 2.5 W/cm^2^ for 10 min. Scale bar is 50 μm.


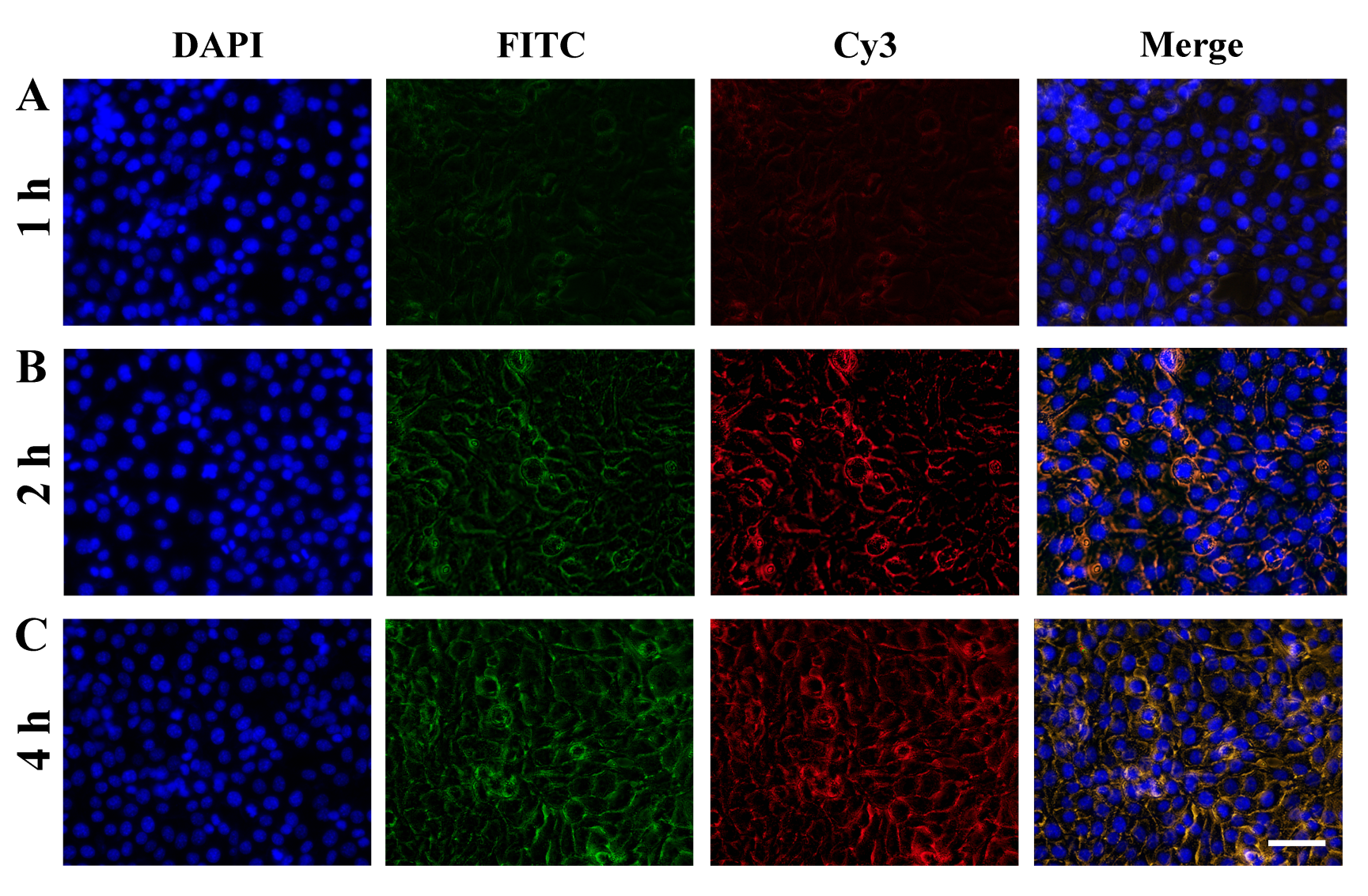


Figure S13. Cellular uptake of CFDTF in 4T1 cells for (A) 1 h, (B) 2 h and (C) 4 h. The cells were incubated with CFDTF for 1, 2 and 4 h and then exposed to 808 nm laser irradiation at the power density of 2.5 W/cm^2^ for 10 min, respectively. Scale bar is 50 μm.


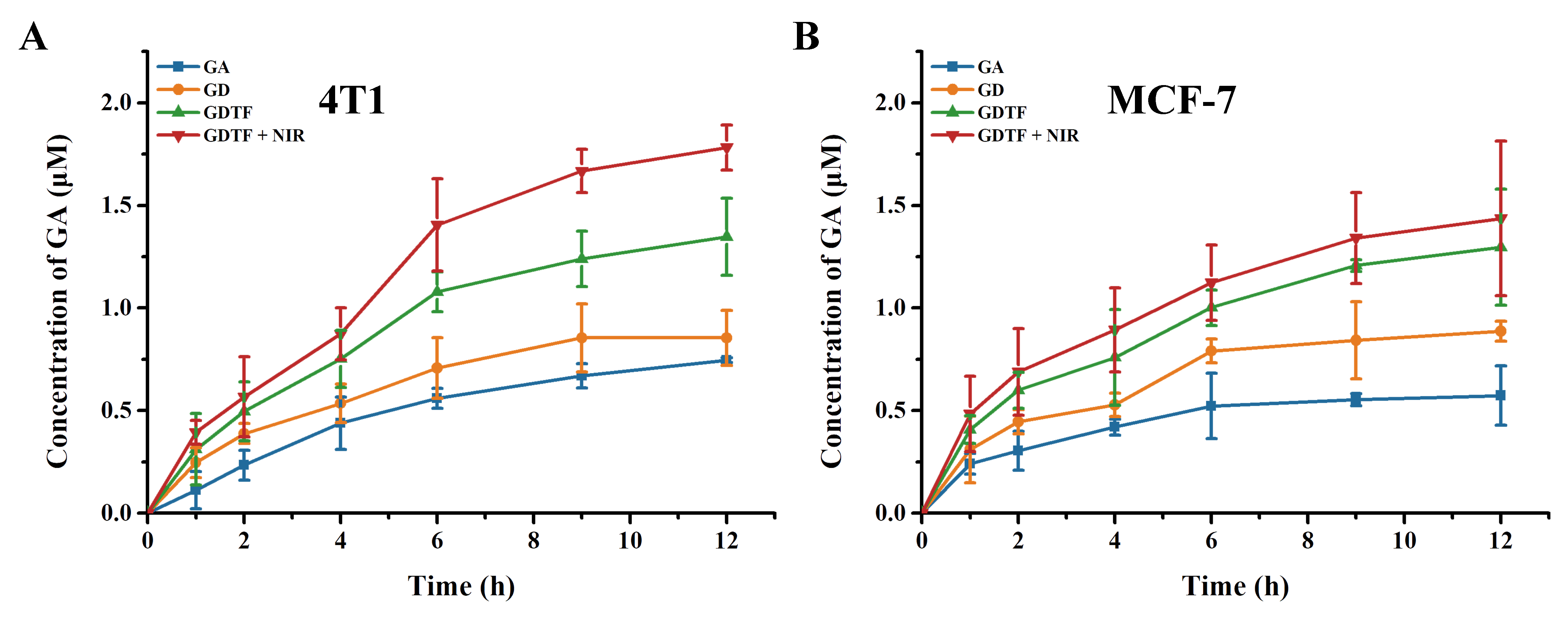


Figure S14. Cellular uptake of GA, GD, GDTF and GDTF + NIR at various time points in (A) 4T1 cells and (B) MCF-7 cells. The cells were incubated with CFDTF for various time points and then exposed to 808 nm laser irradiation at the power density of 2.5 W/cm^2^ for 10 min, respectively (n = 3).


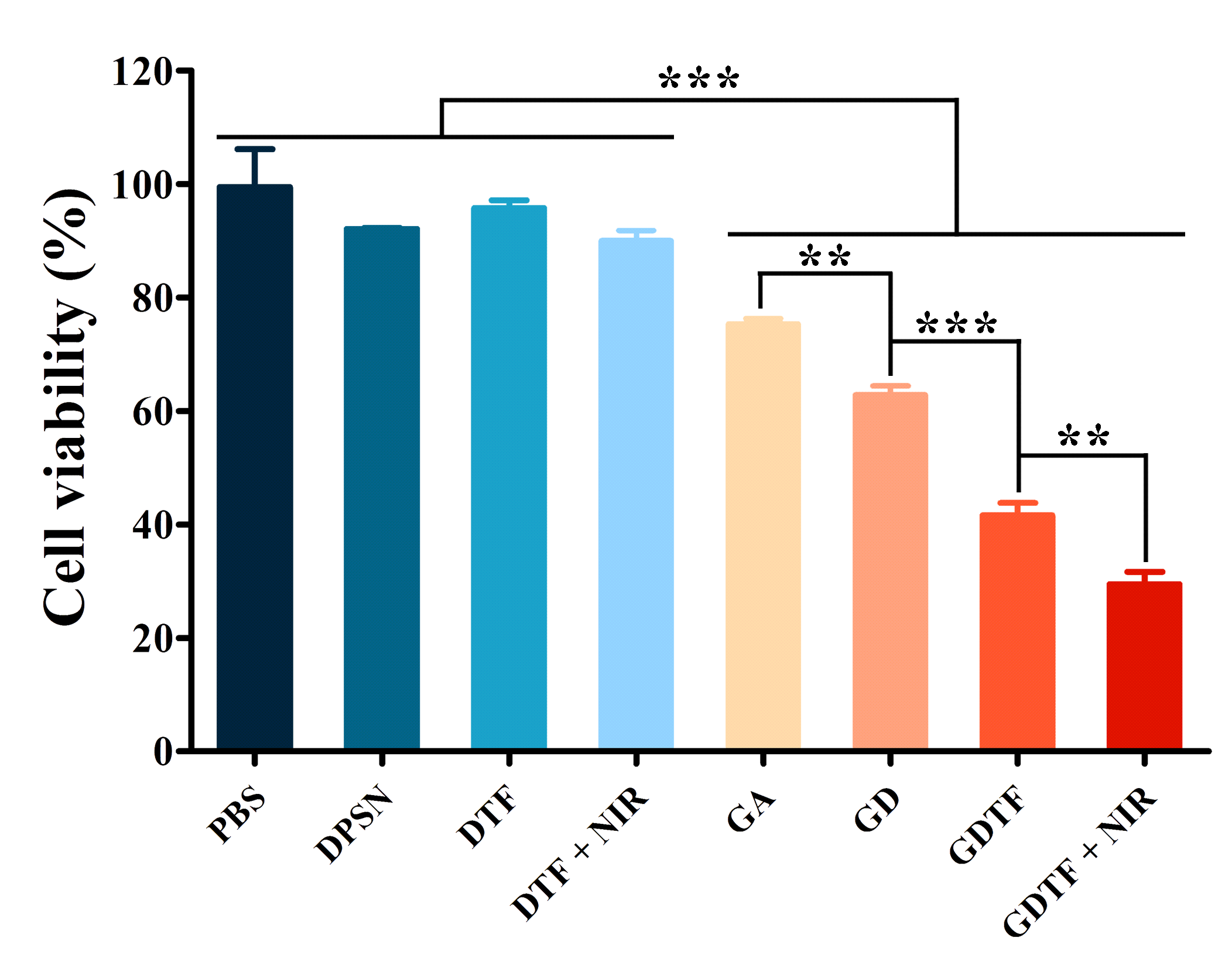


Figure S15. Cell viability of MCF-7 cells after incubation with PBS, DPSN, DTF, GA, GD and GDTF for 24 h. After incubation with DTF and GDTF for 4 h, the cells were exposed to 808 nm laser irradiation at a power density of 2.5 W/cm^2^ for 10 min and then incubated for 20 h. ^**^*P* < 0.01 and ^***^*P* < 0.001, significantly different.


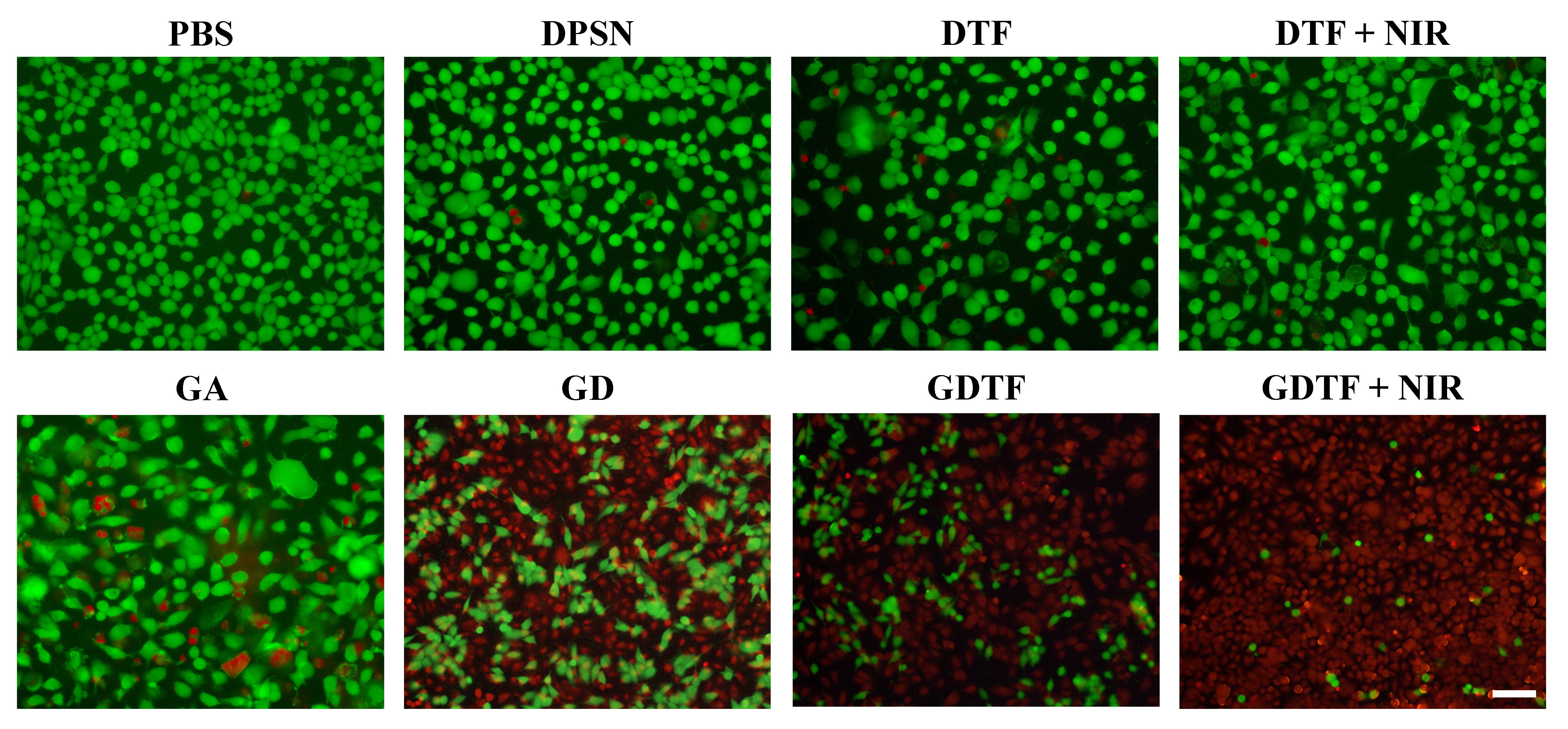


Figure S16. Calcein AM/PI double staining of MCF-7 cells after incubation with PBS, DPSN, DTF, GA, GD and GDTF for 24 h. After incubation with DTF and GDTF for 4 h, the cells were exposed to 808 nm laser irradiation at a power density of 2.5 W/cm^2^ for 10 min and then incubated for 20 h. The scale bar is 50 μm.


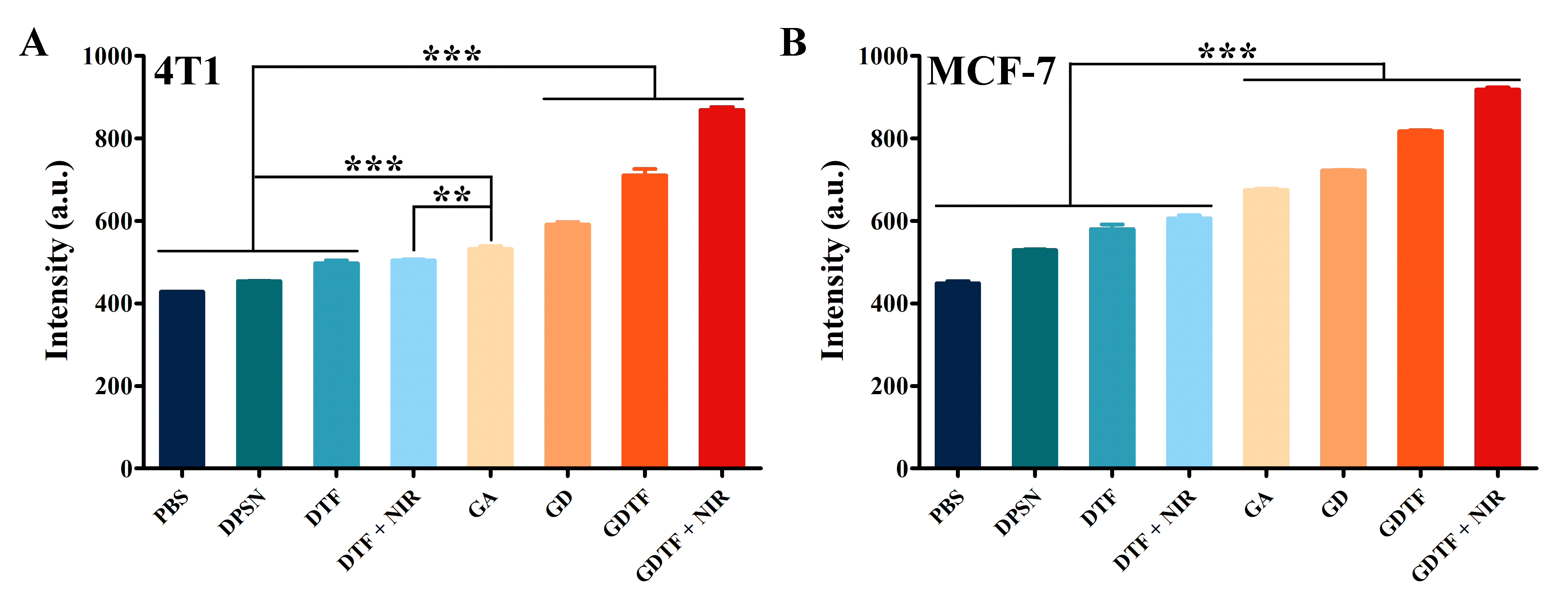


Figure S17. Quantitative analysis for intracellular ROS of (A) 4T1 cells and (B) MCF-7 cells after various treatments. The cells incubated with DTF and GDTF were exposed to 808 nm laser irradiation at the power density of 2.5 W/cm^2^ for 10 min, respectively (n = 3). ^**^*P* < 0.01 and ^***^*P* < 0.001, significantly different.


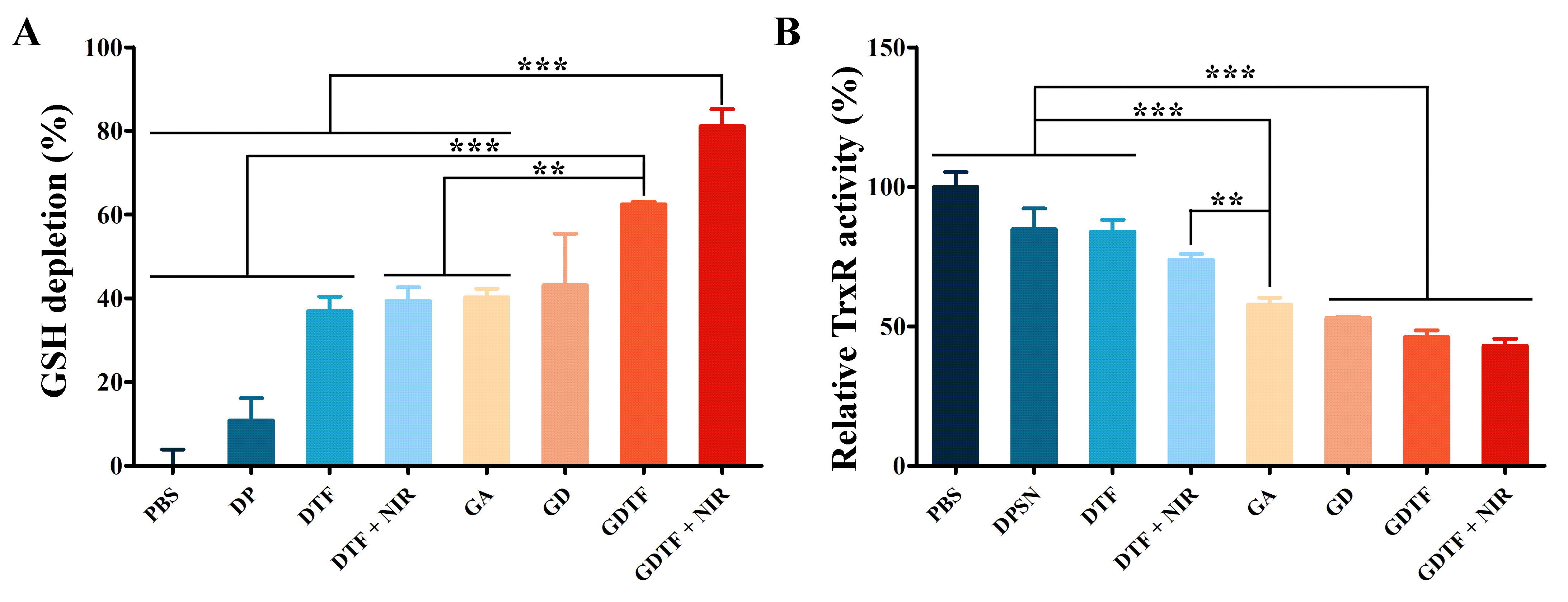


Figure S18. (A) GSH depletion, and (B) relative TrxR activity of MCF-7 cells after incubation with PBS, DPSN, DTF, GA, GD and GDTF for 24 h. ^**^*P* < 0.01 and ^***^*P* < 0.001, significantly different.


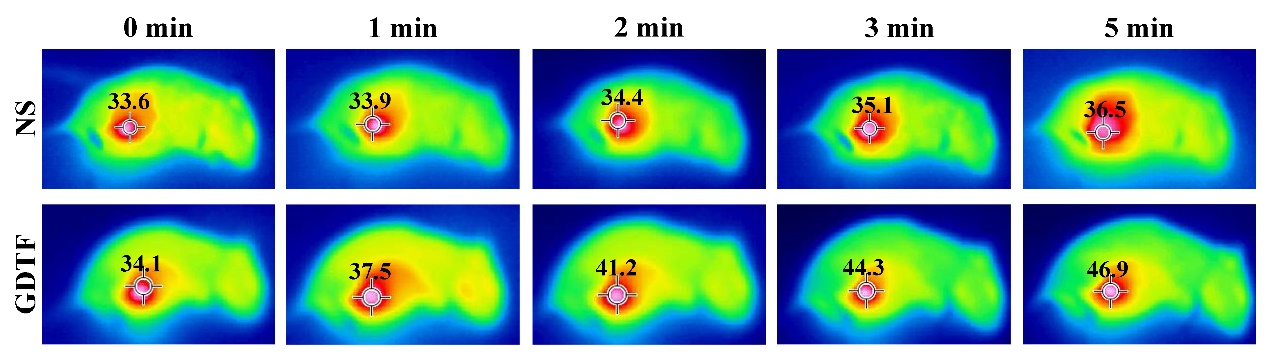


**Figure S19**. *In vivo* near-infrared thermography of the mice injected with NS and GDTF via tail vein. After injection for 4 h, the tumors were exposed to 808 nm laser irradiation at a power density of 1.5 W/cm^2^ for 5 min.


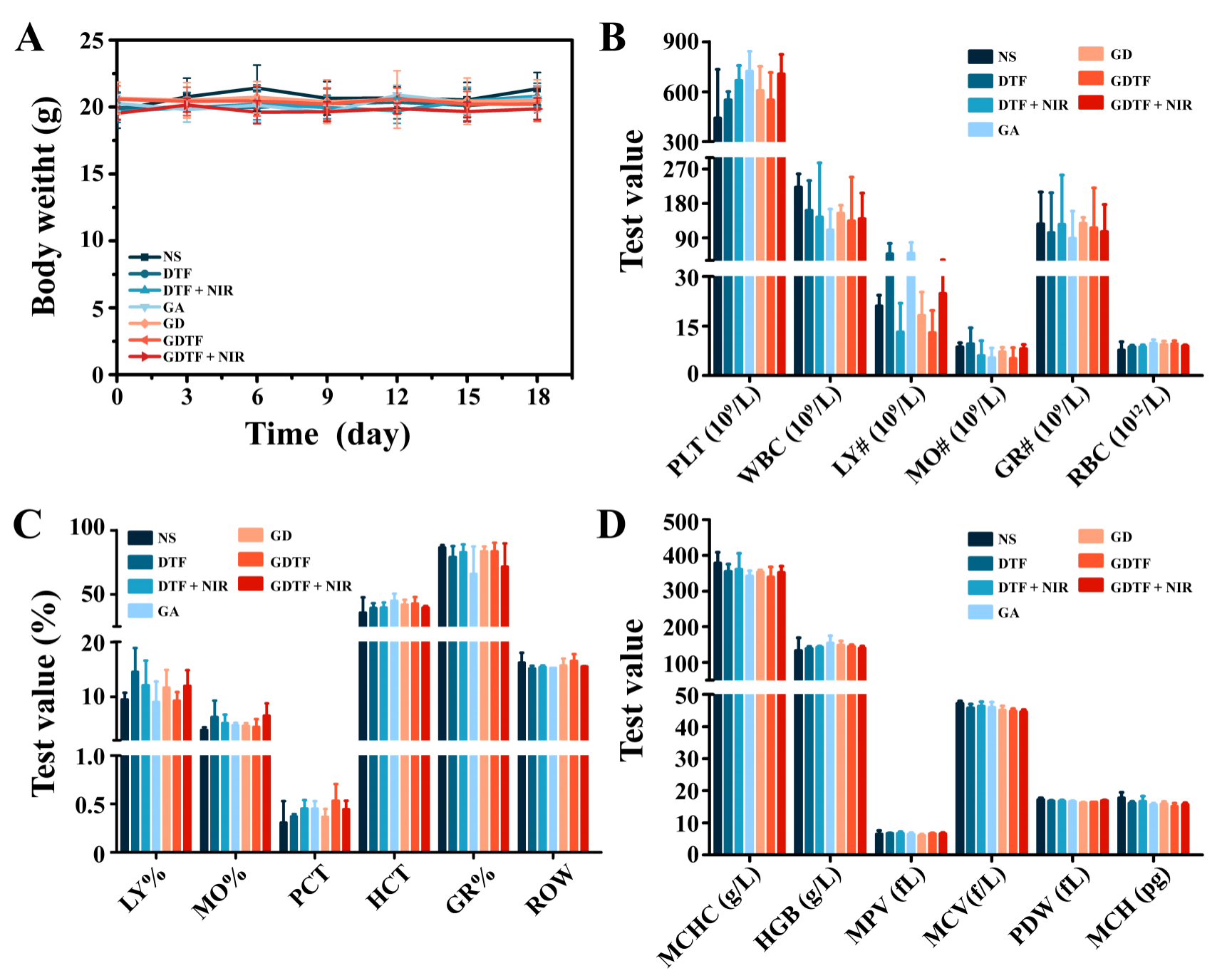


**Figure S20.** Bio-compatibility of GDTF *in vivo*. (A) Body weight change curves of the mice during different treatments. (B-D) Blood routine index examination of the mice after different treatments. All the data were presented as mean ± S.D. (*n* = 3).


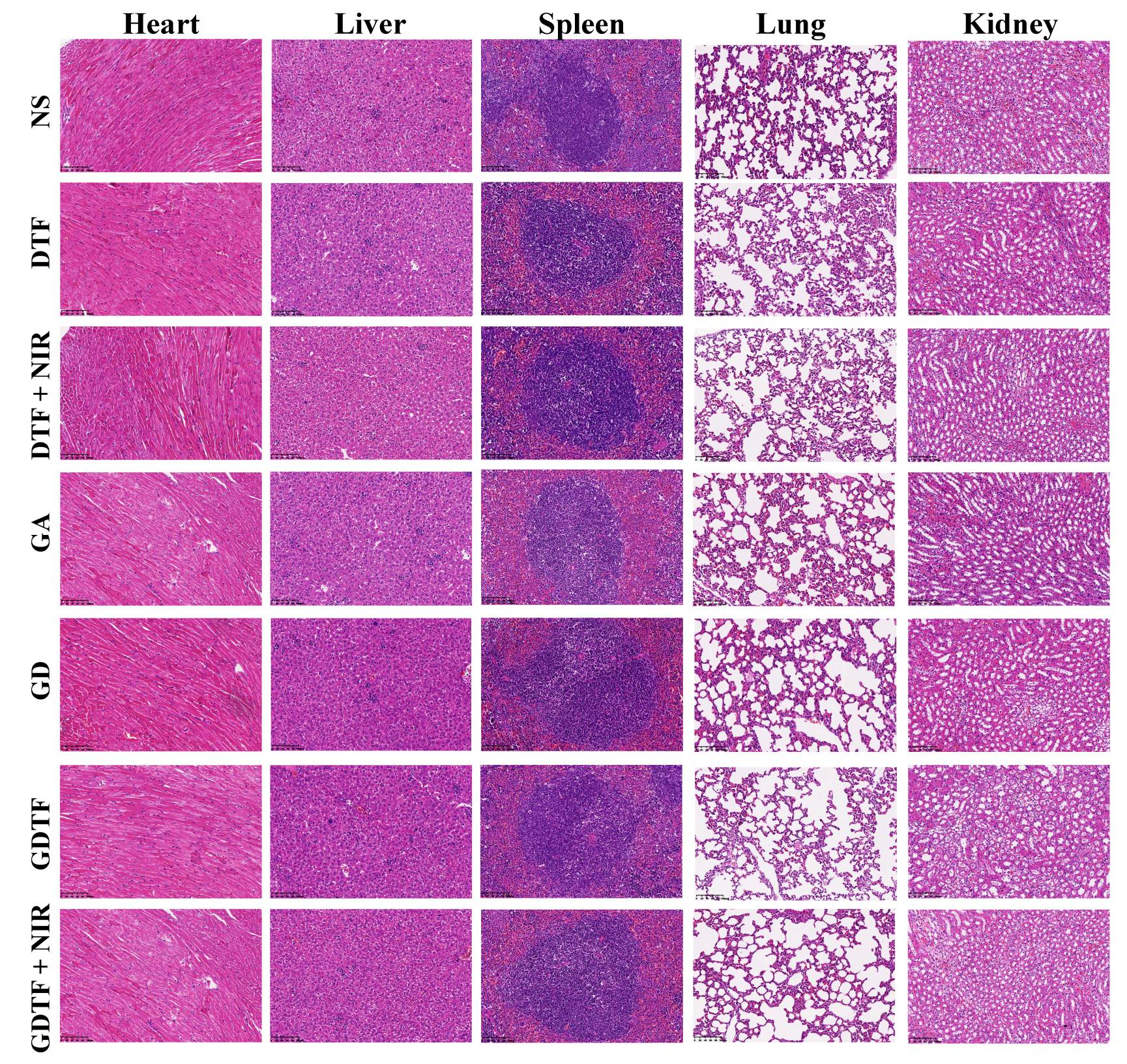


**Figure S21.** H&E staining images of the main organs in the mice after different treatments. The scale bar is 100 μm.

**
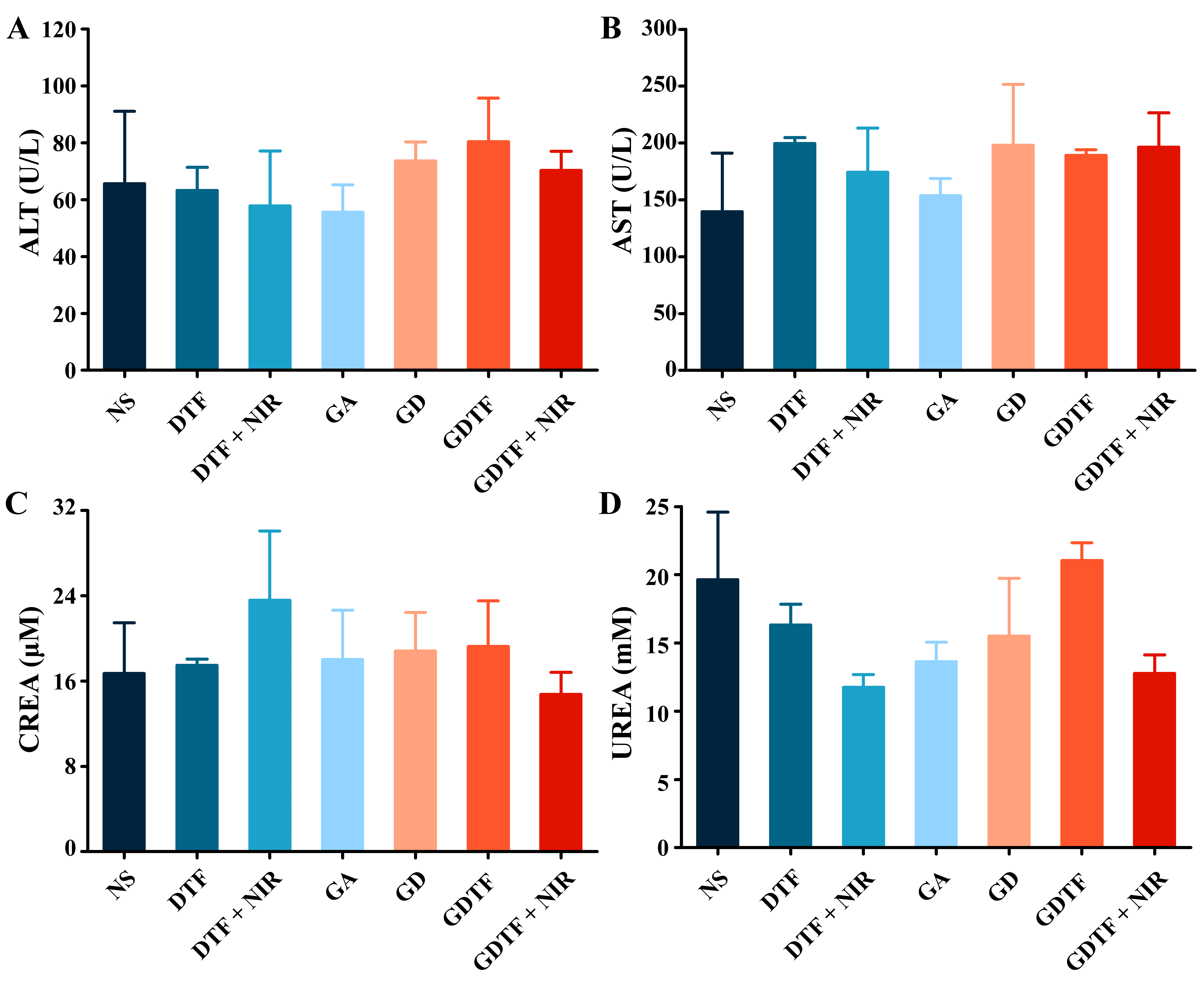
**

**Figure S22.** Serum biochemical index examination including (A) AST, (B) ALT, (C) CREA, and (D) UREA of the mice after different treatments. All the data were presented as mean ± S.D. (*n* = 3).

**Table S1.** The volume of TEOS affected the hydrodynamic size, polydiseperse index (PDI) and zeta potential of DPSN.

| Volume of TEOS (mL) | Hydrodynamic size  (nm) | PDI | Zeta potential  (mV) |
| --- | --- | --- | --- |
| 0.8 | 49.91 ± 0.240 | 0.100 ± 0.010 | -24.8 ± 3.612 |
| 1.0 | 48.87 ± 0.396 | 0.058 ± 0. 018 | -29.7 ± 1.012 |
| 1.2 | 52.24 ± 0.203 | 0.087 ± 0.012 | -22.7 ± 1.429 |

**Table S2.** The template remover affected the hydrodynamic size, PDI and zeta potential of DPSN.

| Template remover | Hydrodynamic size  (nm) | PDI | Zeta potential  (mV) |
| --- | --- | --- | --- |
| 8 mg/mL NaCl in methanol | 102.00 ± 0.458 | 0.319 ± 0.041 | -16.40 ± 0.987 |
| 10% HCl in ethanol | 68.25 ± 0.560 | 0.145 ± 0.005 | -26.47 ± 0.005 |

**Table S3.** The stirring times for the template removal affected the hydrodynamic size, PDI and zeta potential of DPSN.

| Stirring times | Hydrodynamic size  (nm) | PDI | Zeta potential  (mV) |
| --- | --- | --- | --- |
| 1 | 102.0 ± 0.458 | 0.319 ± 0.041 | -16.4 ± 0.987 |
| 3 | 68.25 ± 0.560 | 0.145 ± 0.005 | -26.47 ± 0.005 |
| 5 | 47.89 ± 0.452 | 0.187 ± 0.015 | -27.6 ± 1.044 |

**Table S4.** EE and LD of GD with various mass ratios of drug to carrier.

| Mass ratio of drug to carrier | EE  (%) | DL  (%) |
| --- | --- | --- |
| 2:1 | 1.66 ± 0.12 | 3.21 ± 0.24 |
| 1:1 | 3.60 ± 0.62 | 3.48 ± 0.49 |
| 1:5 | 41.56 ± 2.33 | 7.71 ± 0.42 |
| 1:10 | 97.37 ± 0.38 | 9.16 ± 0.07 |
| 1.20 | 93.71 ± 0.33 | 4.50 ± 0.03 |
